# Impact of CYP and ABCB1 Polymorphisms on Bortezomib-Induced Adverse Events in Multiple Myeloma

**DOI:** 10.3390/biomedicines14040805

**Published:** 2026-04-01

**Authors:** Antonio Sanz-Solas, Noelia Pérez-Gómez, Jorge Labrador, Beatriz Cuevas, María Victoria Cuevas, Francisco Javier Díaz-Gálvez, Gerardo Hermida, Rodolfo Álvarez-Nuño, Gonzalo Benzo, Cristina Alonso-Madrigal, María González-Oter, Natalia García-Sancha, Raquel Vinuesa, Andrea Rodríguez-Lopez, Jesús Novalbos, Natalia Busto, Raquel Alcaraz, Francisco Abad-Santos, Miriam Saiz-Rodríguez

**Affiliations:** 1 Research Unit, Fundación Burgos por la Investigación de la Salud (FBIS), Hospital Universitario de Burgos, 09006 Burgos, Spain; antoniosanz@fbis.org (A.S.-S.); noeliapg@ubu.es (N.P.-G.); jlabradorg@saludcastillayleon.es (J.L.); mgooter@fbis.org (M.G.-O.); nataliags@fbis.org (N.G.-S.); rvinuesa@fbis.org (R.V.); ralcaraz@fbis.org (R.A.); 2 Pharmacology Department, Facultad de Medicina, Universidad Autónoma de Madrid, 28049 Madrid, Spain; francisco.abad@salud.madrid.org; 3 Facultad de Ciencias de la Salud, Universidad de Burgos, 09001 Burgos, Spain; nbusto@ubu.es; 4 Hematology Department, Hospital Universitario de Burgos, 09006 Burgos, Spain; bcuevas@saludcastillayleon.es (B.C.); vcuevas@saludcastillayleon.es (M.V.C.); fcdiaz@saludcastillayleon.es (F.J.D.-G.); ghermida@saludcastillayleon.es (G.H.); ralvarezn@saludcastillayleon.es (R.Á.-N.); 5 Hematology Department, Hospital Universitario de La Princesa, 28006 Madrid, Spain; gonzalo.benzo@salud.madrid.org; 6 Hematology Department, Hospital Santiago Apóstol, Miranda de Ebro, 09200 Burgos, Spain; calonsomadri@saludcastillayleon.es; 7 Cancer Genetics Group, Unit of Excellence Institute of Biomedicine and Molecular Genetics, University of Valladolid Spanish National Research Council (IBGM; UVa-CSIC), 47003 Valladolid, Spain; 8 Clinical Pharmacology Department, Hospital Universitario de La Princesa, Universidad Autónoma de Madrid, Instituto de Investigación Sanitaria La Princesa (IIS-Princesa), 28006 Madrid, Spain; arodriguezl.externo@salud.madrid.org (A.R.-L.); jesus.novalbos@salud.madrid.org (J.N.)

**Keywords:** multiple myeloma, bortezomib, CYP polymorphisms, ABCB1 polymorphisms, adverse drug reactions, pharmacogenomics

## Abstract

**Purpose**: Bortezomib (BTZ) is widely used in multiple myeloma (MM), but its toxicity shows marked interindividual variability. This study aimed to identify pharmacogenetic and clinical factors associated with BTZ-related adverse drug reactions (ADRs). **Methods**: A retrospective and prospective observational study was conducted in 127 MM patients treated with BTZ-based regimens. Polymorphisms in CYP enzymes and ABCB1 were genotyped using qPCR. Associations between genetic variants, treatment response, and ADRs were assessed using univariate and multivariate analyses with Benjamini–Hochberg correction. **Results**: ADRs occurred in 98.4% of patients, most commonly gastrointestinal toxicity (49%), general toxicity (46%), and peripheral neuropathy (39%). Women showed higher rates of gastrointestinal toxicity and non-peripheral neurotoxicity. Multivariate analysis identified the ABCB1 C1236T A/G genotype as protective against gastrointestinal toxicity, while the CYP3A4 intermediate metabolizer phenotype was associated with increased psychiatric toxicity. TP53 mutations were independently associated with hematologic and renal toxicity. Kaplan–Meier analysis showed earlier onset of peripheral neuropathy and respiratory toxicity in CYP3A4 intermediate and poor metabolizers. **Conclusions**: Genetic variation in ABCB1 and CYP3A4, together with clinical factors such as TP53 mutation and sex, may contribute to interindividual variability in BTZ safety in MM. These findings should be considered exploratory given the sample size and require confirmation in larger cohorts. Nonetheless, they suggest a potential role for pharmacogenomics in supporting future approaches to treatment personalization.

## 1. Introduction

Multiple myeloma (MM) is a heterogeneous hematological malignancy characterized by abnormal proliferation of a single clone of plasma cells derived from B cells in the bone marrow [1,2,3]. Currently, induction therapy with bortezomib (BTZ), lenalidomide, and dexamethasone (VRD), followed by autologous stem cell transplantation, consolidation therapy with VRD, and maintenance therapy with lenalidomide are considered standard care for transplantation-eligible patients with newly diagnosed MM [4].

BTZ is a proteasome inhibitor (PI) that reversibly binds to the chymotrypsin-like subunit of the 26S proteasome, the central component of ubiquitin-proteasome system (UPS), thereby preventing the degradation of multiple pro-apoptotic factors [5]. The UPS is a proteolytic system in eukaryotic cells that is responsible for degrading approximately 80–90% of proteins playing a crucial role in transcription, cell cycle regulation, proliferation, signaling, and apoptosis [6]. BTZ was the first PI to be incorporated into clinical practice and continues to be utilized as a first-line treatment for MM and mantle cell lymphoma [7]. Its accumulation will eventually activate the programmed cell death via caspase-mediated pathways in the neoplastic cells that are usually dependent on the suppression of pro-apoptotic pathways for their proliferation and survival [5].

Despite its clinical benefit, BTZ is characterized by a narrow therapeutic index, and an overdose may occur if the dosage is doubled. Under such conditions, patients typically experience significant thrombocytopenia and hypotension, which are notably difficult to reverse [5].

BTZ is known to cause peripheral neuropathy (PN), typically presenting as moderate to severe neuropathic pain in the distal extremities, such as the fingertips, toes, or soles. Patients frequently experience symmetrical numbness and paraesthesia in the hands and feet. Neurological examinations often reveal reduced ankle reflexes and a reduction in distal pinprick and vibration sensitivity. Although motor fibers are generally unaffected, severely affected patients with PN may experience muscle cramps, atrophy, or diminished strength in distal muscles [8,9]. A retrospective analysis of 8218 MM patients enrolled in phase III clinical trials reported a median overall PN incidence of 37.8%, underscoring the substantial burden of BTZ-induced neurotoxicity [10]. This complication often requires a dose modification or changes in the MM treatment plan [11].

Moreover, orthostatic hypotension and gastrointestinal disorders (diarrhea and constipation) have been reported by 12% and nearly 50% of patients, respectively, and more frequently in those patients without overt BTZ-induced PN [9].

Given the marked interpatient variability in the occurrence and severity of PN and other adverse drug reactions (ADRs), research efforts should focus on identifying genetic factors that may predispose certain individuals to increased susceptibility.

Variations in genes encoding drug-metabolizing enzymes and transport proteins can influence the efficacy and toxicity of therapeutic agents. Among the most relevant transporters, ATP-binding cassette (ABC) represent one of the largest protein families in the human genome, involved in the membrane transport of drugs, xenobiotics, endogenous substrates, and ions. Polymorphisms in *ABCB1* gene (which encodes for P-glycoprotein) have been shown to affect drug absorption, distribution, and elimination. Notably, the haplotype consisting of 1236C>T (rs1128503), 2677G>T (rs2032582), and 3435C>T (rs1045642) polymorphisms has been reported to alter substrate specificity of ABCB1 [12,13].

In addition, BTZ is primarily metabolized by cytochrome P450 (CYP) enzymes. CYP3A4 is the main contributor, accounting for approximately 38.4% of its metabolism, followed by CYP2C19 (30.1%) and, to a lesser extent, CYP1A2 (10.5%). CYP2D6 contributes only around 7%, making it unlikely that poor metabolizer phenotypes for this enzyme would significantly affect drug bioavailability [14,15,16,17].

Currently, there is insufficient evidence to support the clinical use of pharmacogenetics for adjusting BTZ therapy in MM patients. Studies exploring genetic polymorphisms in drug-metabolizing enzymes and transporters as potential biomarkers for BTZ efficacy and safety remain limited [18], thus preventing the implementation of personalized treatment strategies based on genetic profiling. Zhou et al. describe no association between CYP2C19 and CYP3A4 activity and BTZ efficacy or the incidence of PN. However, the sample size included in this investigation was too small (n = 56) to identify a significant number of individuals with CYP3A4 decreased function, and it was not clear which variations were employed to construct CYP3A4 metabolizer phenotype [19]. According to our knowledge, this is the first study to explore simultaneously all possible pharmacogenetic biomarkers in the context of BTZ-induced PN and other ADRs in MM patients.

## 2. Materials and Methods

### 2.1. Study Population, Design and Procedures

This retrospective and prospective observational study analyzed the clinical data of patients with MM treated with BTZ. The same inclusion and exclusion criteria were applied across both periods: patients aged ≥18 years, diagnosed with multiple myeloma, receiving at least one cycle of a bortezomib-containing regimen, and with availability of a biological sample for pharmacogenetic analysis. We included patients from the Hematological Department of Hospital Universitario de Burgos (Burgos, Spain), Hospital Universitario de La Princesa (Madrid, Spain) and Hospital Santiago Apóstol (Miranda de Ebro, Burgos, Spain) from May 2022 to April 2025.

The main variables assessed in the study were treatment response evaluated according to the International Uniform Response Criteria for Multiple Myeloma [20], and ADRs, with particular emphasis on PN. The data collected from the medical records included demographic factors (sex, age, weight and height), characteristics of MM (Type Ig, International Staging System (ISS), Revised ISS (R-ISS), cytogenetics, previous pathologies, best response achieved based on the international criteria [20], and adverse events that appeared during the course of treatment with BTZ. This study’s criteria and collected data were defined in accordance with prior clinical and observational studies in MM patients [21]. Treatment regimens, including dose and schedule of bortezomib, were obtained from the clinical records of all participants. All collaborating centers followed the same national therapeutic guidelines, ensuring uniformity in treatment protocols. To minimize heterogeneity across sites, clinical data collection was performed using a standardized protocol, identical for all centers and recruitment periods.

Information on adverse cytogenetic abnormalities, including IGH (immunoglobulin heavy locus) translocations [t(11;14), t(13;14), and t(14;16)] and copy number alterations [1p deletion, 1q gain and del(17p)], was obtained from whole-genome sequencing–based fluorescence in situ hybridization (Seq-FISH) data generated at the Hematology Department, University Hospital of Salamanca, IBSAL, Salamanca, Spain [22].

The primary safety endpoint was the incidence and severity of ADRs. The classification system described in BTZ Summary of Product Characteristics was used to group ADRs, ensuring a consistent classification system across both retrospective and prospective patients [23]. Death from any cause was also recorded. Causality of identified adverse events was determined using the causality algorithm of the Spanish Pharmacovigilance System. Only those categorized as definite, probable, or possible adverse events were considered as ADRs and included in the statistical analysis. In the retrospective cohort, ADRs were extracted from electronic medical records, whereas in the prospective cohort they were systematically assessed during clinical visits. This approach ensured uniformity in ADR identification despite differences in data collection between cohorts.

We also documented the use of concomitant medications known to inhibit or induce CYP3A4, as well as those that inhibit CYP2D6 and CYP2C19. The potential impact of these drug interactions on the study variables was subsequently evaluated.

This study complied with the Declaration of Helsinki and current Spanish legislation on clinical research in humans [24]. It was approved by the Ethics Committee of Drug Research of Health Area of Burgos and Soria, Spain (registry number CEIm-2727).

### 2.2. Genotyping

DNA was extracted from 300 µL of peripheral blood samples using a Maxwell^®^ RSC Whole Blood DNA Kit in an automatic DNA extractor (Maxwell^®^ RSC Instruments) and quantified using a high-precision quantifier Fluorimeter Qubit ™ Flex (Invitrogen, Thermo Fisher Scientific, Waltham, MA, USA).

Forty-five polymorphisms in nine genes of metabolizing enzymes and transporters have been analyzed. A complete list of the analyzed variants and their minor allele frequency is described in Supplementary Table S1. The genotyping was performed by quantitative polymerase chain reaction (qPCR) using a ViiA7^®^ PCR Instrument (Applied Biosystems, Foster City, CA, USA) and customized TaqMan^®^ Array Cards following the manufacturer recommendations (Applied Biosystems, CA, USA). All assays were performed with an internal quality control method with a reproducibility of 100%. *CYP2D6* copy number variation (CNV) was also analyzed by Long-range PCR [25]. Several quality control procedures were implemented to ensure the reliability of the genotyping results, including predefined call-rate thresholds, the use of internal positive and negative controls in each assay, and duplicate genotyping when required.

### 2.3. Statistical Analysis

Genotypes were classified into phenotypes according to Clinical Pharmacogenetics Implementation Consortium (CPIC) allele definition and allele functionality tables [26]. Therefore, individuals were classified into poor metabolizers (PM), intermediate metabolizers (IM), normal metabolizers (NM), rapid metabolizers (RM), and ultrarapid metabolizers (UM) groups. The phenotype assignment of *CYP1A2* genotypes was performed according to the definitions and functional characterizations described previously [27].

All analyses were performed using R statistical software v.4.4.2 (R Core Team, Vienna, Austria) through the RStudio interface (RStudio Team, 2023; version 4.1.1) at the Research Unit of the Hospital Universitario de Burgos. A descriptive and frequency analysis was first conducted. The main outcome variable was the incidence of ADRs. Its relationship with potential predictors including age, sex, weight, body mass index (BMI), race, concomitant treatment, comorbidities, treatment response, and genetic polymorphisms was explored.

Differences in treatment response and genotype frequencies by sex and comparisons of qualitative variables across phenotype groups were assessed using Pearson’s chi-square test or Fisher’s exact test.

Multivariate analyses included step-wise logistic regression to evaluate factors influencing treatment response and ADR incidence, applying Benjamini–Hochberg (pBH) correction to the *p*-values coefficients obtained from the multivariate models to control the false discovery rate. All clinically relevant variables (age, sex, comorbidities, ECOG status, cytogenetic risk), along with all pharmacogenetic variants, were initially entered as candidate predictors in the logistic regression models. A stepwise variable selection procedure based on the Akaike Information Criterion was applied to determine variable retention or removal. The risk of multicollinearity was considered low because the genetic predictors corresponded to independent SNPs analyzed individually. In addition, the AIC-based stepwise selection procedure penalizes redundant predictors, thereby minimizing the likelihood of retaining collinear variables.

Time to event data for the onset of ADRs were analyzed using Kaplan–Meier survival curves, with log-rank tests applied for group comparisons. Statistical significance was set at *p* < 0.05.

## 3. Results

### 3.1. Patient’s Characteristics

A total of 127 patients were included in the study: 93 patients from Hospital Universitario de Burgos (Burgos, Spain), 28 patients from Hospital Universitario de La Princesa (Madrid, Spain) enrolled, and 6 patients from Hospital Santiago Apóstol (Miranda de Ebro, Burgos, Spain). Of these, 99 patients were recruited retrospectively and 28 were recruited prospectively. The main demographic and clinical characteristics of this population are described in Table 1. The study population consisted of 73 men (57%) and 54 women (43%). The mean age was 72 ± 13 years, and the mean BMI was 26.4 ± 3.9. Regarding the distribution of monoclonal component types, IgG was the most common (41%), followed by IgA (28%) and Bence Jones protein (26%). IgM was present in 3.3% of cases, and 0.8% were non-secretors. In four patients, the isotype was unknown.

A total of 13% of the cohort had previously progressed from monoclonal gammopathy of undetermined significance before being diagnosed with MM.

According to the ISS, which was included as a clinical variable in the analyses when available, 49% of patients were classified as stage I, 17% as stage II, and 34% as stage III. However, ISS data were not available for 44 patients, which may have influenced the interpretation of some clinical associations. Since restricting the analysis to patients with complete ISS information would have substantially reduced the sample size and potentially introduced selection bias, the full cohort was retained.

BTZ was used as first-line treatment in 88% of cases. The most frequently used regimen was VRD, administered to 33% of patients. Detailed information on treatment regimens was available for all patients, including dosing schedules. The main variation observed was the administration schedule of bortezomib (weekly vs. twice weekly), reflecting standard clinical practice. Importantly, in all cases, bortezomib was administered subcutaneously. Among the cohort, 39% presented with adverse cytogenetic risk, and 7% were refractory to treatment.

### 3.2. Genotype Frequencies

The corresponding genotypic and phenotypic frequencies of the analyzed genes are presented in Table 2. The genotyping rate was higher than 99% for all the polymorphisms analyzed.

All variants were in Hardy–Weinberg equilibrium, with allele frequencies consistent with those reported for the Iberian (IBS) population in the 1000 Genomes Project [28]. A statistically significant association was observed between CYP1A2 genotypes and sex (*p* = 0.036). Notably, the UM phenotype was more prevalent in males (62%) compared to females (42%) (Table 3). No statistically significant association was observed between other CYP metabolizer phenotype and sex.

No significant associations were found between the pharmacogenetic phenotypes analyzed and the evaluated clinical variables.

### 3.3. Response Achieved

Finally, stable disease (SD) was found in 4.2% of men, while no cases were recorded among women. During treatment with bortezomib, 51 patients achieved a CR (60%) and 23 achieved a CR (27%). No significant association was observed between genetic phenotypes and treatment response in the univariate analysis. However, in the multivariate analysis, the ABCB1 C3435T A/G genotype showed a tendency toward a lower probability of achieving CR (pBH = 0.0539). The concomitant use of CYP enzyme inducers or inhibitors did not exert a statistically significant influence on therapeutic outcomes within the study cohort.

### 3.4. Analysis of Toxicity

Overall, 98.4% of the patients presented at least one adverse event. A total of 361 ADRs were documented, with causality assessed as possible, probable or defined. The most commonly reported toxicities were gastrointestinal toxicity (49%), general toxicity (46%), PN (39%), respiratory toxicity (28%) and cutaneous toxicity (18%). During the treatment period, 17 patients (13%) died as a result of the progression of MM.

Most ADRs occurred at comparable rates between men and women, without statistically significant differences (Table 3). However, two notable exceptions were observed. Gastrointestinal toxicity was significantly more frequent among women compared to men (65% vs. 37%, *p* = 0.002). Likewise, non-peripheral neurotoxicity was also more prevalent in women (15% vs. 2.7%, *p* = 0.018). In addition, there was a trend towards higher overall neurotoxicity in women compared to men (50% vs. 34%, *p* = 0.074), although this did not reach statistical significance. No other ADRs demonstrated meaningful sex-related differences.

In the univariate analysis several genetic variants showed significant associations with specific ADRs. Gastrointestinal toxicity was significantly associated with the three ABCB1 polymorphisms: C1236T (*p* < 0.001), G2677AT (*p* = 0.002), and C3435T (*p* = 0.043). Additionally, ABCB1_C3435T was also linked to respiratory toxicity (*p* = 0.028). Moreover, CYP2C9 PM phenotype was associated with hepatic toxicity (*p* = 0.016) and CYP3A4 IM to psychiatric toxicity (*p* = 0.031). Additional exploratory signals included double-hit MM, associated with respiratory toxicity (*p* = 0.041), and CCND1, which showed borderline associations with hepatic and ocular toxicity (both *p* = 0.062). Mutations in TP53 were associated with hematological (*p* = 0.031), gastrointestinal (*p* = 0.015), ocular (*p* = 0.025), and renal toxicity (*p* = 0.023) (Supplementary Table S2).

In the multivariate logistic regression analysis, several clinical and genetic predictors of complete remission and ADRs remained significant after adjustment. For hematological toxicity, TP53 mutations were associated with an increased risk (pBH = 0.0395). Gastrointestinal toxicity was influenced by multiple factors. The ABCB1 C1236T A/G genotype was associated with a protective effect (pBH = 0.0128), while female sex (pBH = 0.0063) and CYP1A2 UM phenotype (pBH = 0.0379) were both associated with an increased risk. The CYP3A4 IM phenotype was significantly correlated with an increased risk of psychiatric toxicity (pBH = 0.0236). Regarding musculoskeletal toxicity, the ABCB1 C3435T A/G genotype suggested a protective effect (pBH = 0.0616), whereas the use of CYP2D6 inhibitors markedly increased the risk (pBH = 0.0172). Both TP53 mutations and CYP3A5 IM phenotype were significantly associated with an increased risk of renal toxicity, with adjusted *p*-values (BH) of 0.0464 and 0.0471, respectively. Regarding mortality, older age increased risk (pBH = 0.0243) whereas female sex was protective (pBH = 0.0264) (Table 4).

These results highlight the contribution of TP53 alterations and polymorphisms in ABCB1, CYP3A4, and CYP3A5, together with clinical factors such as sex, age, and concomitant CYP2D6 inhibitor use, in shaping the efficacy and safety profile of BTZ therapy in MM.

**Figure 1:**
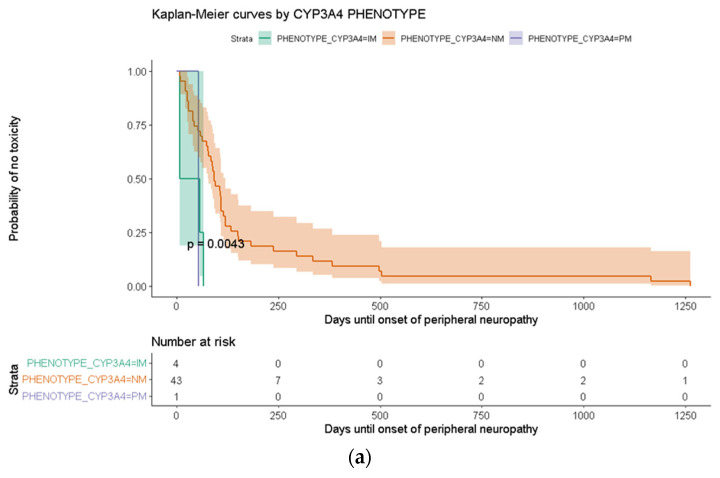

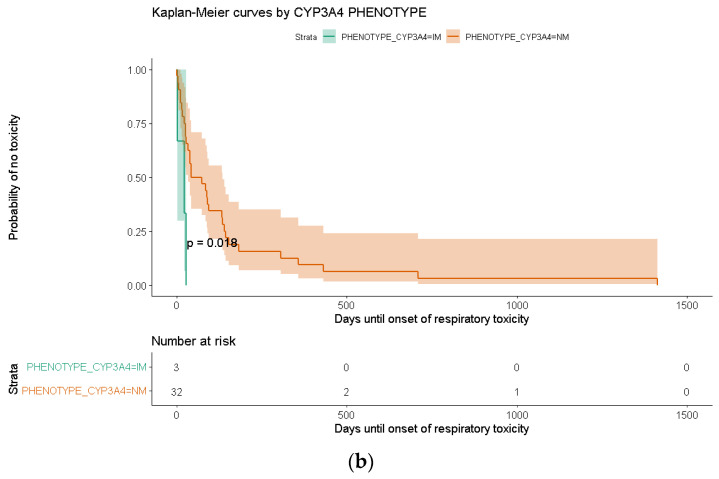
Kaplan–Meier curves of time to onset of toxicity according to CYP3A4 phenotype. (**a**) Time to first occurrence of peripheral neuropathy stratified by CYP3A4 phenotype; (**b**) Time to first occurrence of respiratory toxicity stratified by CYP3A4 phenotype.

Kaplan–Meier analysis was performed to evaluate the influence of CYP3A4 phenotypes on the time to onset of ADRs. Patients with IM and PM phenotypes developed PN significantly earlier compared to those with the NM phenotype (*p* = 0.0043) (Figure 1a). Similarly, the respiratory toxicity occurred earlier in IM individuals relative to NM patients (*p* = 0.018) (Figure 1b).

Detailed descriptive statistics from the Kaplan–Meier analyses, including the mean time to event and corresponding 95% IC, are available in Supplementary Table S3.

To control for potential confounding effects related to concomitant therapies, treatment with lenalidomide and dexamethasone was also analyzed. No significant associations were observed between the use of lenalidomide or dexametasone and treatment response or the incidence of ADRs in the multivariate models, suggesting that the observed pharmacogenetic effects were independent of these concomitant therapies.

## 4. Discussion

Despite its proven efficacy, BTZ therapy is frequently limited by interindividual variability in drug response and toxicity, which can compromise treatment adherence and clinical outcomes [29,30]. MM often requires intensive treatment regimens that include proteasome inhibitors such as BTZ. While BTZ has significantly improved survival outcomes in MM, its use is associated with several ADRs, including PN, gastrointestinal, hematologic, and musculoskeletal toxicities. Evidence suggests that interindividual differences in drug metabolism and transport, influenced by genetic variants, contribute substantially to this variability. These observations provide a rationale for exploring pharmacogenomic profiling as a tool to predict both efficacy and toxicity. In this context, our findings indicate that interindividual variability in BTZ response and toxicity is primarily driven by genes involved in drug metabolism and transport, rather than by intrinsic MM genetic factors, although these observations should be considered exploratory due to the limited subgroup sizes.

Our cohort has shown demographic, disease and treatment characteristics consistent with prior MM registries [31,32,33]. The expected phenotype frequencies are consistent with those reported in the European population [18]. However, we observed a higher prevalence of CYP1A2 UM among men compared to women. This finding may be a cohort-specific random variation, as it has not been previously documented in the literature.

The percentage of patients who achieved a treatment response in our cohort was comparable to that reported in previous clinical trials and observational studies of BTZ-based regimens in MM [34]. Regarding treatment response, no significant associations were found in the univariate analysis, whereas the multivariate model revealed a borderline trend toward reduced complete response rates in carriers of the *ABCB1* C3435T A/G genotype. This finding may indicate a potential modulatory role of ABCB1 in intracellular drug exposure; however, given the borderline significance and sample size, this association should be viewed as preliminary and requires further validation. Previous pharmacogenetic studies in MM have also reported a limited influence of *ABCB1* SNPs (Single Nucleotide Polymorphism) on lenalidomide response, emphasizing the necessity of larger studies to reliably identify strong predictive effects within this gene [35]. Notably, in our study, no significant effect of concomitant lenalidomide or dexamethasone treatment was observed, suggesting that the pharmacogenetic associations identified were independent of these therapies. Our lack of results related the association of CYP enzymes phenotypes and treatment response differs from the results reported by Goel et al., who described an association between the CYP2C19*2 allele and response to bortezomib-based therapy [36]. Nevertheless, this discrepancy does not undermine the relevance of pharmacogenetic variability in bortezomib treatment, but rather highlights the heterogeneity of interindividual drug response, where different metabolic and transport pathways may predominate across distinct patient populations. Together, these observations support the concept that response to bortezomib is influenced by multiple genetic determinants with context-dependent effects.

Almost all patients showed at least one ADR, consistent with the known toxicity of proteasome inhibitors. The most prevalent toxicities; gastrointestinal, general, and PN are in line with prior clinical observations described in the literature [37]. When stratified by sex, women exhibited significantly higher rates of gastrointestinal toxicity and non-peripheral neurotoxicity. The multivariate analysis confirmed that female sex increased the risk of gastrointestinal toxicity. Previous studies have similarly reported a higher incidence of ADRs among women treated with anticancer agents, potentially linked to sex-related differences in drug metabolism and slower clearance rates, resulting in increased systemic exposure [38,39,40].

**Pharmacogenetic findings and clinical predictors.**

Univariate analyses revealed associations between all three *ABCB1* SNPs (C1236T rs1128503, G2677AT rs2032582, and C3435T rs1045642) and gastrointestinal toxicity. These findings are biologically plausible because *ABCB1* encodes P-glycoprotein, a key efflux transporter expressed in intestinal and hematopoietic tissues; however, the extent to which these polymorphisms alter BTZ distribution or local exposure remains uncertain, and the observed associations should be interpreted as preliminary. Variants in *ABCB1* have been reported to modify P-glycoprotein expression or function, potentially influencing mucosal drug concentrations and thereby contributing to gastrointestinal adverse events. Consistently, in the multivariate gastrointestinal toxicity model, the *ABCB1* C1236T A/G genotype remained nominally protective, suggesting a functional impact of P-glycoprotein variability on treatment tolerability. These results are in line with the previous study of Gudur et al., 2024, which reported that *ABCB1* C1236T and C3435T polymorphisms were associated with adriamycin or paclitaxel and induced gastrointestinal toxicity in breast cancer, supporting the notion that genetic variation in *ABCB1* can influence drug exposure and toxicity in clinical settings [41]. Nevertheless, given the moderate sample size of our cohort, the protective association observed for the C1236T A/G genotype should be interpreted with caution and considered exploratory.

Additionally, the CYP1A2 UM phenotype was associated with an increased risk of gastrointestinal toxicity. This suggests that secondary metabolic pathways mediated by CYP1A2 may contribute to gastrointestinal toxicity, beyond the CYP3A4-mediated metabolism of BTZ. Nonetheless, this hypothesis is speculative and requires confirmation in mechanistic studies or larger datasets.

The CYP3A4 IM phenotype was independently associated with psychiatric toxicity supporting the idea that reduced enzymatic activity and slower clearance may enhance central nervous system exposure. Although bortezomib is not predominantly metabolized by CYP3A4, the enzyme contributes to secondary metabolic pathways that may influence systemic drug handling or the formation of neurotoxic metabolites. These mechanistic hypotheses remain insufficiently characterized, and the association observed in our study requires confirmation in larger cohorts. Even though PN was one of the most relevant and frequent toxicities in the cohort, no significant association was detected in the multivariate model. However, Kaplan–Meier analyses showed that CYP3A4 IM or PM phenotypes developed PN and respiratory toxicity significantly earlier than NM, supporting a kinetic model in which slower metabolism prolongs systemic exposure and accelerates ADRs onset. The potential influence of CYP3A4 inhibition was also evaluated, but concomitant use of CYP3A4 inhibitors did not show a significant association with any ADR. In line with this, renal toxicity was independently associated with the CYP3A5 IM phenotype. Given its overlapping substrate specificity with CYP3A4, reduced CYP3A5 activity may alter metabolite disposition, renal clearance, or local drug accumulation, potentially contributing to increased renal toxicity. These results raise the possibility that CYP3A4-mediated metabolism plays a role in both the intensity and timing of BTZ-induced ADRs, although the limited number of patients in certain phenotype groups warrants a cautious interpretation. Given that BTZ undergoes partial CYP-mediated biotransformation, these associations are biologically plausible, but the specific metabolites or pathways involved remain insufficiently characterized. This mechanism has also been observed with other CYP3A4-metabolized drugs, such as statins, where reduced enzymatic activity leads to prolonged systemic exposure and an increased risk of early or more severe toxicity. These findings are consistent with a kinetic model in which slower CYP3A4 metabolism enhances drug accumulation and toxicity, coherent with evidence reported for multiple CYP3A substrates [42,43]. However, in interpreting our pharmacogenetic findings, it is important to acknowledge that several variants of interest were represented by very small genotype groups, including CYP3A4. This limited representation restricts the robustness of the statistical analyses and may contribute to unstable or spurious associations. For this reason, we respectfully request that these findings be interpreted with due caution, recognizing their primarily hypothesis-generating nature. Future research with larger cohorts or pooled datasets will be essential to validate the potential associations observed in this study.

Notably, the association between use of CYP2D6 inhibitors and musculoskeletal toxicity likely reflects drug–drug interactions resulting in elevated BTZ levels or metabolite accumulation in muscle tissue. The combined administration of enzyme inhibitors may alter and increase toxicity due to poor elimination [44]. However, as CYP2D6 is not the primary enzyme involved in BTZ metabolism, further studies are needed to confirm this association.

Finally, *TP53* mutations were associated with hematologic, gastrointestinal, ocular, and renal toxicities in univariate analysis, and remained independently predictive of hematologic and renal toxicity in multivariate models. While TP53 is not directly involved in drug metabolism, its mutation is linked to more aggressive disease and poorer prognosis in hematologic malignancies [22,45]. Given that MM frequently involves renal impairment as part of its clinical manifestation, it is plausible that more severe disease driven by *TP53* mutations contributes to the increased renal and hematologic toxicity observed in our cohort [46,47]. Thus, the observed associations may reflect disease-related vulnerability.

Importantly, some of the associations identified in our study were observed only in univariate analyses and did not remain significant in multivariate models, suggesting that they may be influenced by confounding clinical factors. For this reason, we respectfully suggest that these findings be appraised with appropriate caution, given their exploratory and hypothesis-generating nature.

**Implications for personalized therapy.**

Overall, our results are consistent with the pharmacologic profile of BTZ, which is primarily processed through CYP450 enzymes and modulated by drug transporters. The observed involvement of *ABCB1* and of *CYP3A4* variants in determining drug transport and elimination rates aligns with emerging evidence in MM pharmacogenetics [18]. The TP53 associations likely reflect underlying disease severity rather than pharmacokinetic modulation, as this mutation is linked to more aggressive MM and increased baseline organ vulnerability. Altogether, this pharmacogenomic study underscores the need for integrative models combining metabolism, transport, and clinical factors to predict BTZ tolerability. Nevertheless, these observations should not yet be extrapolated to clinical decision-making, as their mechanistic basis remains incomplete and the associations require validation in larger prospective cohorts.

## 5. Future Perspectives and Limitations

This study provides novel insights into the pharmacogenetic determinants of BTZ toxicity in MM. However, certain limitations should be acknowledged.

The sample size may have limited the detection of associations with smaller effect sizes, particularly for rare genotypes, and therefore these findings should be interpreted with caution. A major limitation of this study is the very small number of patients in certain genotype subgroups, especially those carrying rare pharmacogenetic variants, which substantially reduces statistical power and increases the likelihood of chance findings. Moreover, multicollinearity among predictors was not formally assessed, which may have affected the stability of the multivariate models. Consequently, results derived from these low-frequency subgroups should be considered exploratory and require validation in larger cohorts or through collaborative multicenter analyses to confirm these preliminary observations. Although ISS was included in the analyses whenever available and showed no significant association with ADRs, the missing data may have influenced the interpretation of some clinical correlations. Additionally, the retrospective design and the heterogeneity in treatment regimens could introduce potential confounders. To mitigate this, patients were analyzed according to their specific treatment protocols, and this stratified approach helped reduce variability without revealing significant differences between groups. Despite these limitations, the findings highlight clinically relevant biomarkers that warrant further investigation. Future multicenter and prospective studies with larger cohorts and integrated pharmacokinetic analyses are needed to confirm these associations and to establish the clinical utility of pharmacogenetic testing for guiding BTZ therapy in MM.

## 6. Conclusions

This study identifies key pharmacogenetic and clinical factors influencing BTZ safety and efficacy in MM. Variants in ABCB1, CYP3A4, and CYP3A5, along with sex, age, and concomitant CYP2D6 inhibitor use, were found to modulate the risk and timing of specific ADRs. These results suggest a potential role of drug-metabolizing enzymes and transporters in determining interindividual variability. The influence of TP53 mutations on hematologic and renal toxicity reflects the aggressiveness of the disease rather than direct pharmacokinetic modulation. Overall, these findings align with the pharmacologic profile of BTZ, mainly metabolized by CYP450 enzymes and influenced by transporters that regulate its distribution and elimination. The findings suggest a possible incorporation of pharmacogenomic profiling into clinical decision-making to optimize BTZ dosing and minimize toxicity. Nonetheless, larger, multicenter, and prospective studies are required to validate these associations, elucidate underlying mechanisms, and confirm the clinical utility of these biomarkers in routine practice.

## Figures and Tables

**Table 1:** Demographic, clinical, and genetic characteristics of the study population.

Characteristic		N = 127
Sex		
	Men	73 (57%)
	Women	54 (43%)
Age		72 ± 13
BMI		26.4 ± 3.9
Age at diagnosis		67 ± 12
Type Ig	A	35 (28%)
	BJ	32 (26%)
	G	51 (41%)
	M	4 (3.3%)
	non-secretory	1 (0.8%)
Adverse cytogenetics (n = 92)		36 (39%)
1q gain		16 (13%)
Del(17p) TP53		9 (7.1%)
t(14;16) FGFR3		7 (5.5%)
t(11;14) MAF		1 (0.8%)
1p deletion		2 (1.6%)
t(13;14) CCND1		2 (1.6%)
MM doble hit		8 (6.3%)
1st line treatment (n = 117)		103 (88%)
Refractory (n = 122)		9 (7%)

Abbreviations: BMI (Body Mass Index), Ig (Inmunoglobulins), BJ (Bence Jones), Del (deletion), TP53 (tumor protein p53), FGFR3 (fibroblast growth factor receptor 3), MAF (musculoaponeurotic fibrosarcoma), CCND1 (Cyclin D1), t (traslocation) and MM (multiple myeloma).

**Table 2:** Distribution of pharmacogenetic variants and metabolic phenotypes by sex in the study population.

Gene	Genotype/Phenotype	Overall	Men	Women	*p*-Value
		N = 127	n = 73	n = 54	
ABCB1_C1236T rs1128503	A/A	22 (17%)	12 (16%)	10 (19%)	0.8
	A/G	59 (47%)	36 (49%)	23 (43%)	
	G/G	45 (36%)	25 (34%)	20 (38%)	
ABCB1_C3435T rs1045642	A/A	22 (17%)	10 (14%)	12 (23%)	0.3
	A/G	68 (54%)	43 (59%)	25 (47%)	
	G/G	36 (29%)	20 (27%)	16 (30%)	
ABCB1_G2677AT rs2032582	A/A	16 (13%)	8 (11%)	8 (15%)	0.6
	C/A_C/T	63 (50%)	39 (53%)	24 (45%)	
	C/C	47 (37%)	26 (36%)	21 (40%)	
CYP1A2	PM	1 (0.8%)	0 (0%)	1 (1.9%)	0.036
	NM	58 (46%)	28 (38%)	30 (57%)	
	UM	67 (53%)	45 (62%)	22 (42%)	
CYP2B6	PM	15 (12%)	10 (14%)	5 (9.4%)	0.7
	IM	47 (37%)	29 (40%)	18 (34%)	
	NM	59 (47%)	31 (42%)	28 (53%)	
	RM	5 (4.0%)	3 (4.1%)	2 (3.8%)	
CYP2C19	PM	1 (0.8%)	0 (0%)	1 (1.9%)	0.9
	IM	32 (25%)	18 (25%)	14 (26%)	
	NM	60 (48%)	35 (48%)	25 (47%)	
	RM	29 (23%)	17 (23%)	12 (23%)	
	UM	4 (3.2%)	3 (4.1%)	1 (1.9%)	
CYP2C9	PM	4 (3.2%)	3 (4.1%)	1 (1.9%)	0.3
	IM	57 (45%)	29 (40%)	28 (53%)	
	NM	65 (52%)	41 (56%)	24 (45%)	
CYP2D6	PM	9 (7.1%)	7 (9.6%)	2 (3.8%)	0.2
	IM	41 (33%)	22 (30%)	19 (36%)	
	NM	69 (55%)	42 (58%)	27 (51%)	
	UM	7 (5.6%)	2 (2.7%)	5 (9.4%)	
CYP3A4	PM	1 (0.8%)	0 (0%)	1 (1.9%)	0.5
	IM	10 (7.9%)	5 (6.8%)	5 (9.4%)	
	NM	115 (91%)	68 (93%)	47 (89%)	
CYP3A5	PM	111 (88%)	63 (86%)	48 (91%)	0.5
	IM	15 (12%)	10 (14%)	5 (9.4%)	
TP53	Mutated	9 (7.1%)	5 (6.8%)	4 (7.4%)	0.9

Abbreviations: ABCB1 (ATP binding cassette subfamily B member 1), CYP (Cytochrome P-450), PM (poor metabolizer), IM (intermediate metabolizer), NM (normal metabolizer), RM (rapid metabolizer) and UM (ultrarapid metabolizer).

**Table 3:** Adverse drug reactions stratified by sex in multiple myeloma patients treated with bortezomib.

Type of Adverse Drug Reaction	Overall	Men	Women	*p*-Value
	N = 127	N = 73	N = 54	
Hematological	11 (8.7%)	6 (8.2%)	5 (9.3%)	>0.9
Gastrointestinal	62 (49%)	27 (37%)	35 (65%)	0.002
Respiratory	36 (28%)	24 (33%)	12 (22%)	0.2
Neurotoxicity	52 (41%)	25 (34%)	27 (50%)	0.074
Peripheral neuropathy	49 (39%)	24 (33%)	25 (46%)	0.12
Neurotoxicity different from peripheral neuropathy	10 (7.9%)	2 (2.7%)	8 (15%)	0.018
Infections	7 (5.5%)	4 (5.5%)	3 (5.6%)	>0.9
Metabolic toxicity and nutrition	1 (0.8%)	0 (0%)	1 (1.9%)	0.4
Psychiatric	9 (7.1%)	3 (4.1%)	6 (11%)	0.2
General	58 (46%)	32 (44%)	26 (48%)	0.6
Ocular	4 (3.1%)	1 (1.4%)	3 (5.6%)	0.3
Ototoxicity	3 (2.4%)	1 (1.4%)	2 (3.7%)	0.6
Cardiotoxicity	4 (3.1%)	2 (2.7%)	2 (3.7%)	>0.9
Vascular toxicity	4 (3.1%)	3 (4.1%)	1 (1.9%)	0.6
Hepatotoxicity	4 (3.1%)	1 (1.4%)	3 (5.6%)	0.3
Musculoskeletal	13 (10%)	7 (9.6%)	6 (11%)	0.8
Cutaneous	23 (18%)	14 (19%)	9 (17%)	0.7
Nephrotoxicity	10 (7.9%)	8 (11%)	2 (3.7%)	0.2
Reproductive toxicity	1 (0.8%)	1 (1.4%)	0 (0%)	>0.9

Adverse reactions (% patients with ADR).

**Table 4:** Results from multivariate analysis.

**Complete remission**					
**Variable**	**Odds Ratio 95%**	**95% CI lower**	**95% CI upper**	***p*-Value**	**pBH**
ABCB1 C3435T A/G	0.1414	0.0322	5.01 × 10^−1^	0.0045	0.0539
**Hematologic toxicity**					
**Variable**	**Odds Ratio 95%**	**95% CI lower**	**95% CI upper**	***p*-Value**	**pBH**
P53 Mut	5.875	1.0864	2.72 × 10^1^	0.0264	0.0395
**Gastrointestinal toxicity**					
**Variable**	**Odds Ratio 95%**	**95% CI lower**	**95% CI upper**	***p*-Value**	**pBH**
ABCB1 C1236T A/G	2.23 × 10^−1^	0.0784	5.86 × 10^−1^	0.0032	0.0128
Sex Fem	4.5816	1.9358	11.5826	0.0008	0.0063
CYP1A2 UM	3.24 × 10^0^	1.3101	8.6786	0.0142	0.0379
**Psychiatric toxicity**					
**Variable**	**Odds Ratio 95%**	**95% CI lower**	**95% CI upper**	***p*-Value**	**pBH**
CYP3A4 IM	1.53 × 10^1^	2.09	1.40 × 10^2^	0.0079	0.0236
**Musculoskeletal toxicity**					
**Variable**	**Odds Ratio 95%**	**95% CI lower**	**95% CI upper**	***p*-Value**	**pBH**
ABCB1 C3435T A/G	0.1346	0.0207	7.56 × 10^−1^	0.0264	0.0616
CYP2D6 inhibitors	2.14 × 10^1^	2.6512	2.14 × 10^4^	0.0049	0.0172
**Renal toxicity**					
**Variable**	**Odds Ratio 95%**	**95% CI lower**	**95% CI upper**	***p*-Value**	**pBH**
P53 Mut	10.0315	1.4777	69.2371	0.0153	0.0464
CYP3A5 IM	7.70 × 10^0^	1.2399	4.80 × 10^1^	0.0235	0.0471
**Exitus**					
**Variable**	**Odds Ratio 95%**	**95% CI lower**	**95% CI upper**	***p*-Value**	***p*-Value-Value (BH adj)**
Age	1.08 × 10^0^	1.0231	1.14 × 10^0^	0.0081	0.0243
Sex Fem	1.52 × 10^−1^	0.0271	5.85 × 10^−1^	0.0132	0.0264

Abbreviations: ABCB1 (ATP binding cassette subfamily B member 1), CYP (Cytochrome P-450), IM (intermediate metabolizer), UM (ultrarapid metabolizer).

## Data Availability

The original contributions presented in the study are included in the article and Supplementary Material; further inquiries can be directed to the corresponding author.

## Supplementary Materials

The following supporting information can be downloaded at: https://www.mdpi.com/article/10.3390/biomedicines14040805/s1.
