# Supplementary material for: Impact of CYP and ABCB1 Polymorphisms on Bortezomib-Induced Adverse Events in Multiple Myeloma
Source: Biomedicines. 2026 Apr 1;14(4):805. doi: 10.3390/biomedicines14040805 (PMC13112948; doi:10.3390/biomedicines14040805)
Supplement: Supplementary file 1 [file biomedicines-14-00805-s001.zip › biomedicines-4210819-supplementary.pdf]

Supplementary Table S1: Summary of polymorphisms in CYP and *ABCB1* genes potentially involved in BTZ pharmacokinetics and their frequencies in the European population.

**Gene**	**Polymorphism**	**Reference allele**	**Alternative allele**	**European MAF**
CYP3A4	*3 rs4986910	A	G	0,007
CYP3A4	*2 rs55785340	A	G/T	0,002
CYP3A4	*6 rs4646438	T	TT	0
CYP3A4	*18 rs28371759	A	G	0
CYP3A4	*20 rs67666821	T	DEL	<0.1
CYP3A4	*22 rs35599367	G	A	0,05
CYP3A5	*3 rs776746	T	C	0,943
CYP3A5	*6 rs10264272	C	T	0,003
CYP3A5	*7 rs41303343	A	AA	0
CYP2C19	*2 rs4244285	G	A/C	0,145
CYP2C19	*3 rs4986893	G	A	0
CYP2C19	*4 rs28399504	A	G/T	0,001
CYP2C19	*17 rs12248560	C	A/T	0,224
CYP1A2	*1C rs2069514	G	A	0,02
CYP1A2	*1F rs762551	C	A/G	0,68
CYP1A2	*1B rs2470890	T	C	0,404
CYP2D6	*3 rs35742686	T	DEL	0,019
CYP2D6	*4 rs3892097	C	T	0,186
CYP2D6	*6 rs5030655	UN	DEL	0,02
CYP2D6	*7 rs5030867	T	G	0
CYP2D6	*8 rs5030865	C	A/G/T	0
CYP2D6	*9 rs5030656	CTTCT	CT (INDEL)	0,026
CYP2D6	*10 rs1065852	G	A/C	0,173
CYP2D6	*10 rs1135840	C	G	0,546
CYP2D6	*12 rs5030862	C	T	0
CYP2D6	*14 rs5030865	C	A/G/T	0
CYP2D6	*15 rs774671100	A	AA	0,000385
CYP2D6	*17 rs28371706	G	C/T	0,002
CYP2D6	*19 rs72549353	AGTTAG	AG	0,00015
CYP2D6	*29 rs59421388	C	T	0
CYP2D6	*41 rs28371725	C	T	0,093
CYP2D6	*56B rs72549347	G	A	0
CYP2D6	*59 rs79292917	C	T	0,002
CYP2D6	*12*45*46 rs28371710	C	T	0
CYP2D6	CNV (*5 - deletion, duplication)			
CYP2B6	*4 rs2279343	A	G	0,135
CYP2B6	*5 rs3211371	C	T	0,098
CYP2B6	*9 rs3745274	G	T	0,206

Gene	Polymorphism	Reference allele	Alternative allele	European MAF
CYP2C9	*2 rs1799853	C	T	0,124
CYP2C9	*3 rs1057910	A	C/G	0,073
CYP2C9	*5 rs28371686	C	**A**/G	0
CYP2C9	*8 rs9332094	T	C	0,001
CYP2C9	*8 rs7900194	G	A/C/T	0,002
CYP2C9	*11 rs28371685	C	T	0,002
ABCB1	C3435T rs1045642	A	C/G/T	0,482
ABCB1	C1236T rs1128503	A	G	0,584
ABCB1	G2677T/A rs2032582	C	TA	0,573

Supplementary Table S2: Incidence of ADRs according to genotype/phenotype

	Haematological				Gastrointestinal				General				Respiratory			
Genotype/Phenotype	No	Yes	Total	p-value[1]	No	Yes	Total	p-value[2]	No	Yes	Total	p-value[2]	No	Yes	Total	p-value[2]
*ABCB1*_C1236T (rs1128503)				0.6				**<0.001**				0.3				0.2
A/A	21 (95%)	1 (4.5%)	22 (100%)		6 (27%)	16 (73%)	22 (100%)		15 (68%)	7 (32%)	22 (100%)		19 (86%)	3 (14%)	22 (100%)	
A/G	52 (88%)	7 (12%)	59 (100%)		41 (69%)	18 (31%)	59 (100%)		30 (51%)	29 (49%)	59 (100%)		42 (71%)	17 (29%)	59 (100%)	
G/G	42 (93%)	3 (6.7%)	45 (100%)		18 (40%)	27 (60%)	45 (100%)		23 (51%)	22 (49%)	45 (100%)		30 (67%)	15 (33%)	45 (100%)	
*ABCB1*_C3435T (rs1045642)				0.3				**0.043**				0.6				**0.028**
A/A	19 (86%)	3 (14%)	22 (100%)		8 (36%)	14 (64%)	22 (100%)		14 (64%)	8 (36%)	22 (100%)		20 (91%)	2 (9.1%)	22 (100%)	
A/G	61 (90%)	7 (10%)	68 (100%)		42 (62%)	26 (38%)	68 (100%)		35 (51%)	33 (49%)	68 (100%)		43 (63%)	25 (37%)	68 (100%)	
G/G	35 (97%)	1 (2.8%)	36 (100%)		15 (42%)	21 (58%)	36 (100%)		19 (53%)	17 (47%)	36 (100%)		28 (78%)	8 (22%)	36 (100%)	
*ABCB1*_G2677AT (rs2032582)				0.3				**0.002**				0.4				0.3
A/A	15 (94%)	1 (6.3%)	16 (100%)		4 (25%)	12 (75%)	16 (100%)		11 (69%)	5 (31%)	16 (100%)		14 (88%)	2 (13%)	16 (100%)	
C/A_C/T	55 (87%)	8 (13%)	63 (100%)		42 (67%)	21 (33%)	63 (100%)		33 (52%)	30 (48%)	63 (100%)		43 (68%)	20 (32%)	63 (100%)	
C/C	45 (96%)	2 (4.3%)	47 (100%)		19 (40%)	28 (60%)	47 (100%)		24 (51%)	23 (49%)	47 (100%)		34 (72%)	13 (28%)	47 (100%)	
CYP1A2				>0.9				0.3				0.5				0.7

	Haematological				Gastrointestinal				General				Respiratory			
Genotype/Phenotype	No	Yes	Total	p-value[1]	No	Yes	Total	p-value[2]	No	Yes	Total	p-value[2]	No	Yes	Total	p-value[2]
NM	53 (91%)	5 (8.6%)	58 (100%)		33 (57%)	25 (43%)	58 (100%)		30 (52%)	28 (48%)	58 (100%)		40 (69%)	18 (31%)	58 (100%)	
PM	1 (100%)	0 (0%)	1 (100%)		0 (0%)	1 (100%)	1 (100%)		0 (0%)	1 (100%)	1 (100%)		1 (100%)	0 (0%)	1 (100%)	
UM	61 (91%)	6 (9.0%)	67 (100%)		32 (48%)	35 (52%)	67 (100%)		38 (57%)	29 (43%)	67 (100%)		50 (75%)	17 (25%)	67 (100%)	
CYP2B6				0.12				0.3				0.7				>0.9
IM	43 (91%)	4 (8.5%)	47 (100%)		28 (60%)	19 (40%)	47 (100%)		26 (55%)	21 (45%)	47 (100%)		35 (74%)	12 (26%)	47 (100%)	
NM	54 (92%)	5 (8.5%)	59 (100%)		28 (47%)	31 (53%)	59 (100%)		30 (51%)	29 (49%)	59 (100%)		42 (71%)	17 (29%)	59 (100%)	
PM	15 (100%)	0 (0%)	15 (100%)		8 (53%)	7 (47%)	15 (100%)		8 (53%)	7 (47%)	15 (100%)		10 (67%)	5 (33%)	15 (100%)	
RM	3 (60%)	2 (40%)	5 (100%)		1 (20%)	4 (80%)	5 (100%)		4 (80%)	1 (20%)	5 (100%)		4 (80%)	1 (20%)	5 (100%)	
CYP2C19				0.5				0.6				0.5				0.3
IM	30 (94%)	2 (6.3%)	32 (100%)		19 (59%)	13 (41%)	32 (100%)		20 (63%)	12 (38%)	32 (100%)		20 (63%)	12 (38%)	32 (100%)	
NM	52 (87%)	8 (13%)	60 (100%)		29 (48%)	31 (52%)	60 (100%)		32 (53%)	28 (47%)	60 (100%)		44 (73%)	16 (27%)	60 (100%)	
PM	1 (100%)	0 (0%)	1 (100%)		0 (0%)	1 (100%)	1 (100%)		0 (0%)	1 (100%)	1 (100%)		1 (100%)	0 (0%)	1 (100%)	
RM	28 (97%)	1 (3.4%)	29 (100%)		14 (48%)	15 (52%)	29 (100%)		15 (52%)	14 (48%)	29 (100%)		24 (83%)	5 (17%)	29 (100%)	
UM	4 (100%)	0 (0%)	4 (100%)		3 (75%)	1 (25%)	4 (100%)		1 (25%)	3 (75%)	4 (100%)		2 (50%)	2 (50%)	4 (100%)	
CYP2C9				0.2				0.2				0.2				0.8

	Haematological				Gastrointestinal				General				Respiratory			
Genotype/Phenotype	No	Yes	Total	p-value[1]	No	Yes	Total	p-value[2]	No	Yes	Total	p-value[2]	No	Yes	Total	p-value[2]
IM	49 (86%)	8 (14%)	57 (100%)		26 (46%)	31 (54%)	57 (100%)		30 (53%)	27 (47%)	57 (100%)		43 (75%)	14 (25%)	57 (100%)	
NM	62 (95%)	3 (4.6%)	65 (100%)		38 (58%)	27 (42%)	65 (100%)		34 (52%)	31 (48%)	65 (100%)		45 (69%)	20 (31%)	65 (100%)	
PM	4 (100%)	0 (0%)	4 (100%)		1 (25%)	3 (75%)	4 (100%)		4 (100%)	0 (0%)	4 (100%)		3 (75%)	1 (25%)	4 (100%)	
CYP2D6				0.2				>0.9				0.9				0.061
IM	38 (93%)	3 (7.3%)	41 (100%)		22 (54%)	19 (46%)	41 (100%)		23 (56%)	18 (44%)	41 (100%)		28 (68%)	13 (32%)	41 (100%)	
NM	64 (93%)	5 (7.2%)	69 (100%)		35 (51%)	34 (49%)	69 (100%)		38 (55%)	31 (45%)	69 (100%)		47 (68%)	22 (32%)	69 (100%)	
PM	8 (89%)	1 (11%)	9 (100%)		4 (44%)	5 (56%)	9 (100%)		4 (44%)	5 (56%)	9 (100%)		9 (100%)	0 (0%)	9 (100%)	
UM	5 (71%)	2 (29%)	7 (100%)		4 (57%)	3 (43%)	7 (100%)		3 (43%)	4 (57%)	7 (100%)		7 (100%)	0 (0%)	7 (100%)	
CYP3A4				0.3				0.6				0.6				>0.9
IM	8 (80%)	2 (20%)	10 (100%)		6 (60%)	4 (40%)	10 (100%)		6 (60%)	4 (40%)	10 (100%)		7 (70%)	3 (30%)	10 (100%)	
NM	106 (92%)	9 (7.8%)	115 (100%)		59 (51%)	56 (49%)	115 (100%)		62 (54%)	53 (46%)	115 (100%)		83 (72%)	32 (28%)	115 (100%)	
PM	1 (100%)	0 (0%)	1 (100%)		0 (0%)	1 (100%)	1 (100%)		0 (0%)	1 (100%)	1 (100%)		1 (100%)	0 (0%)	1 (100%)	
CYP3A5				0.4				0.2				0.6				0.6
IM	15 (100%)	0 (0%)	15 (100%)		10 (67%)	5 (33%)	15 (100%)		9 (60%)	6 (40%)	15 (100%)		12 (80%)	3 (20%)	15 (100%)	

	Haematological				Gastrointestinal				General				Respiratory			
Genotype/Phenotype	No	Yes	Total	p-value[1]	No	Yes	Total	p-value[2]	No	Yes	Total	p-value[2]	No	Yes	Total	p-value[2]
PM	100 (90%)	11 (9.9%)	111 (100%)		55 (50%)	56 (50%)	111 (100%)		59 (53%)	52 (47%)	111 (100%)		79 (71%)	32 (29%)	111 (100%)	
Gain.1p				>0.9				0.2				0.7				0.071
No	101 (91%)	10 (9.0%)	111 (100%)		59 (53%)	52 (47%)	111 (100%)		61 (55%)	50 (45%)	111 (100%)		83 (75%)	28 (25%)	111 (100%)	
Yes	15 (94%)	1 (6.3%)	16 (100%)		6 (38%)	10 (63%)	16 (100%)		8 (50%)	8 (50%)	16 (100%)		8 (50%)	8 (50%)	16 (100%)	
P53				**0.031**				**0.015**				0.7				0.12
No	110 (93%)	8 (6.8%)	118 (100%)		64 (54%)	54 (46%)	118 (100%)		65 (55%)	53 (45%)	118 (100%)		87 (74%)	31 (26%)	118 (100%)	
Yes	6 (67%)	3 (33%)	9 (100%)		1 (11%)	8 (89%)	9 (100%)		4 (44%)	5 (56%)	9 (100%)		4 (44%)	5 (56%)	9 (100%)	
FGFR3				>0.9				0.7				0.7				0.10
No	109 (91%)	11 (9.2%)	120 (100%)		62 (52%)	58 (48%)	120 (100%)		66 (55%)	54 (45%)	120 (100%)		88 (73%)	32 (27%)	120 (100%)	
Yes	7 (100%)	0 (0%)	7 (100%)		3 (43%)	4 (57%)	7 (100%)		3 (43%)	4 (57%)	7 (100%)		3 (43%)	4 (57%)	7 (100%)	
MAF				>0.9				0.5				>0.9				0.3
No	115 (91%)	11 (8.7%)	126 (100%)		65 (52%)	61 (48%)	126 (100%)		68 (54%)	58 (46%)	126 (100%)		91 (72%)	35 (28%)	126 (100%)	
Yes	1 (100%)	0 (0%)	1 (100%)		0 (0%)	1 (100%)	1 (100%)		1 (100%)	0 (0%)	1 (100%)		0 (0%)	1 (100%)	1 (100%)	
Delection.1q				>0.9				0.2				0.2				0.5
No	114 (91%)	11 (8.8%)	125 (100%)		65 (52%)	60 (48%)	125 (100%)		69 (55%)	56 (45%)	125 (100%)		90 (72%)	35 (28%)	125 (100%)	
Yes	2 (100%)	0 (0%)	2 (100%)		0 (0%)	2 (100%)	2 (100%)		0 (0%)	2 (100%)	2 (100%)		1 (50%)	1 (50%)	2 (100%)	

	Haematological				Gastrointestinal				General				Respiratory			
Genotype/Phenotype	No	Yes	Total	p-value[1]	No	Yes	Total	p-value[2]	No	Yes	Total	p-value[2]	No	Yes	Total	p-value[2]
CCND1				0.2				>0.9				>0.9				0.079
No	115 (92%)	10 (8.0%)	125 (100%)		64 (51%)	61 (49%)	125 (100%)		68 (54%)	57 (46%)	125 (100%)		91 (73%)	34 (27%)	125 (100%)	
Yes	1 (50%)	1 (50%)	2 (100%)		1 (50%)	1 (50%)	2 (100%)		1 (50%)	1 (50%)	2 (100%)		0 (0%)	2 (100%)	2 (100%)	
MM.double.hit				0.14				0.2				0.14				**0.041**
No	110 (92%)	9 (7.6%)	119 (100%)		63 (53%)	56 (47%)	119 (100%)		67 (56%)	52 (44%)	119 (100%)		88 (74%)	31 (26%)	119 (100%)	
Yes	6 (75%)	2 (25%)	8 (100%)		2 (25%)	6 (75%)	8 (100%)		2 (25%)	6 (75%)	8 (100%)		3 (38%)	5 (63%)	8 (100%)	

[1]Fisher's exact test

[2]Pearson's Chi-squared test

Incidence of ADRs according to genotype/phenotype

	Neurotoxicity				Neurotoxicity different from peripheral neuropathy				Peripheral neuropathy				Infections			
Genotype/Phenotype	No	Yes	Total	p-value[1]	No	Yes	Total	p-value[2]	No	Yes	Total	p-value[1]	No	Yes	Total	p-value[2]
*ABCB1*_C1236T (rs1128503)				0.6				0.8				0.4				0.3
A/A	15 (68%)	7 (32%)	22 (100%)		20 (91%)	2 (9.1%)	22 (100%)		16 (73%)	6 (27%)	22 (100%)		22 (100%)	0 (0%)	22 (100%)	
A/G	34 (58%)	25 (42%)	59 (100%)		55 (93%)	4 (6.8%)	59 (100%)		36 (61%)	23 (39%)	59 (100%)		56 (95%)	3 (5.1%)	59 (100%)	
G/G	25 (56%)	20 (44%)	45 (100%)		41 (91%)	4 (8.9%)	45 (100%)		25 (56%)	20 (44%)	45 (100%)		41 (91%)	4 (8.9%)	45 (100%)	
*ABCB1*_C3435T (rs1045642)				0.8				0.7				0.6				0.5
A/A	12 (55%)	10 (45%)	22 (100%)		21 (95%)	1 (4.5%)	22 (100%)		12 (55%)	10 (45%)	22 (100%)		22 (100%)	0 (0%)	22 (100%)	
A/G	42 (62%)	26 (38%)	68 (100%)		61 (90%)	7 (10%)	68 (100%)		44 (65%)	24 (35%)	68 (100%)		63 (93%)	5 (7.4%)	68 (100%)	
G/G	20 (56%)	16 (44%)	36 (100%)		34 (94%)	2 (5.6%)	36 (100%)		21 (58%)	15 (42%)	36 (100%)		34 (94%)	2 (5.6%)	36 (100%)	
*ABCB1*_G2677AT (rs2032582)				0.8				0.6				0.6				0.6
A/A	10 (63%)	6 (38%)	16 (100%)		16 (100%)	0 (0%)	16 (100%)		10 (63%)	6 (38%)	16 (100%)		16 (100%)	0 (0%)	16 (100%)	
C/A_C/T	38 (60%)	25 (40%)	63 (100%)		57 (90%)	6 (9.5%)	63 (100%)		41 (65%)	22 (35%)	63 (100%)		60 (95%)	3 (4.8%)	63 (100%)	
C/C	26 (55%)	21 (45%)	47 (100%)		43 (91%)	4 (8.5%)	47 (100%)		26 (55%)	21 (45%)	47 (100%)		43 (91%)	4 (8.5%)	47 (100%)	

	Neurotoxicity				Neurotoxicity different from peripheral neuropathy				Peripheral neuropathy				Infections			
Genotype/Phenotype	No	Yes	Total	p-value[1]	No	Yes	Total	p-value[2]	No	Yes	Total	p-value[1]	No	Yes	Total	p-value[2]
CYP1A2				0.4				0.3				0.8				>0.9
NM	31 (53%)	27 (47%)	58 (100%)		51 (88%)	7 (12%)	58 (100%)		34 (59%)	24 (41%)	58 (100%)		55 (95%)	3 (5.2%)	58 (100%)	
PM	1 (100%)	0 (0%)	1 (100%)		1 (100%)	0 (0%)	1 (100%)		1 (100%)	0 (0%)	1 (100%)		1 (100%)	0 (0%)	1 (100%)	
UM	42 (63%)	25 (37%)	67 (100%)		64 (96%)	3 (4.5%)	67 (100%)		42 (63%)	25 (37%)	67 (100%)		63 (94%)	4 (6.0%)	67 (100%)	
CYP2B6				0.7				0.2				0.4				>0.9
IM	29 (62%)	18 (38%)	47 (100%)		40 (85%)	7 (15%)	47 (100%)		32 (68%)	15 (32%)	47 (100%)		44 (94%)	3 (6.4%)	47 (100%)	
NM	33 (56%)	26 (44%)	59 (100%)		57 (97%)	2 (3.4%)	59 (100%)		34 (58%)	25 (42%)	59 (100%)		56 (95%)	3 (5.1%)	59 (100%)	
PM	8 (53%)	7 (47%)	15 (100%)		14 (93%)	1 (6.7%)	15 (100%)		7 (47%)	8 (53%)	15 (100%)		14 (93%)	1 (6.7%)	15 (100%)	
RM	4 (80%)	1 (20%)	5 (100%)		5 (100%)	0 (0%)	5 (100%)		4 (80%)	1 (20%)	5 (100%)		5 (100%)	0 (0%)	5 (100%)	
CYP2C19				0.7				0.2				0.9				>0.9
IM	16 (50%)	16 (50%)	32 (100%)		27 (84%)	5 (16%)	32 (100%)		18 (56%)	14 (44%)	32 (100%)		30 (94%)	2 (6.3%)	32 (100%)	
NM	38 (63%)	22 (37%)	60 (100%)		58 (97%)	2 (3.3%)	60 (100%)		39 (65%)	21 (35%)	60 (100%)		56 (93%)	4 (6.7%)	60 (100%)	
PM	1 (100%)	0 (0%)	1 (100%)		1 (100%)	0 (0%)	1 (100%)		1 (100%)	0 (0%)	1 (100%)		1 (100%)	0 (0%)	1 (100%)	
RM	17 (59%)	12 (41%)	29 (100%)		26 (90%)	3 (10%)	29 (100%)		17 (59%)	12 (41%)	29 (100%)		28 (97%)	1 (3.4%)	29 (100%)	
UM	2 (50%)	2 (50%)	4 (100%)		4 (100%)	0 (0%)	4 (100%)		2 (50%)	2 (50%)	4 (100%)		4 (100%)	0 (0%)	4 (100%)	

	Neurotoxicity				Neurotoxicity different from peripheral neuropathy				Peripheral neuropathy				Infections			
Genotype/Phenotype	No	Yes	Total	p-value[1]	No	Yes	Total	p-value[2]	No	Yes	Total	p-value[1]	No	Yes	Total	p-value[2]
CYP2C9				0.7				0.8				0.6				0.8
IM	35 (61%)	22 (39%)	57 (100%)		53 (93%)	4 (7.0%)	57 (100%)		37 (65%)	20 (35%)	57 (100%)		53 (93%)	4 (7.0%)	57 (100%)	
NM	36 (55%)	29 (45%)	65 (100%)		59 (91%)	6 (9.2%)	65 (100%)		37 (57%)	28 (43%)	65 (100%)		62 (95%)	3 (4.6%)	65 (100%)	
PM	3 (75%)	1 (25%)	4 (100%)		4 (100%)	0 (0%)	4 (100%)		3 (75%)	1 (25%)	4 (100%)		4 (100%)	0 (0%)	4 (100%)	
CYP2D6				0.9				0.8				0.9				0.091
IM	25 (61%)	16 (39%)	41 (100%)		37 (90%)	4 (9.8%)	41 (100%)		26 (63%)	15 (37%)	41 (100%)		41 (100%)	0 (0%)	41 (100%)	
NM	39 (57%)	30 (43%)	69 (100%)		64 (93%)	5 (7.2%)	69 (100%)		41 (59%)	28 (41%)	69 (100%)		64 (93%)	5 (7.2%)	69 (100%)	
PM	5 (56%)	4 (44%)	9 (100%)		8 (89%)	1 (11%)	9 (100%)		5 (56%)	4 (44%)	9 (100%)		8 (89%)	1 (11%)	9 (100%)	
UM	5 (71%)	2 (29%)	7 (100%)		7 (100%)	0 (0%)	7 (100%)		5 (71%)	2 (29%)	7 (100%)		6 (86%)	1 (14%)	7 (100%)	
CYP3A4				0.6				0.6				0.6				0.5
IM	6 (60%)	4 (40%)	10 (100%)		9 (90%)	1 (10%)	10 (100%)		6 (60%)	4 (40%)	10 (100%)		9 (90%)	1 (10%)	10 (100%)	
NM	68 (59%)	47 (41%)	115 (100%)		106 (92%)	9 (7.8%)	115 (100%)		71 (62%)	44 (38%)	115 (100%)		109 (95%)	6 (5.2%)	115 (100%)	
PM	0 (0%)	1 (100%)	1 (100%)		1 (100%)	0 (0%)	1 (100%)		0 (0%)	1 (100%)	1 (100%)		1 (100%)	0 (0%)	1 (100%)	
CYP3A5				0.5				>0.9				0.3				>0.9
IM	10 (67%)	5 (33%)	15 (100%)		14 (93%)	1 (6.7%)	15 (100%)		11 (73%)	4 (27%)	15 (100%)		15 (100%)	0 (0%)	15 (100%)	

	Neurotoxicity				Neurotoxicity different from peripheral neuropathy				Peripheral neuropathy				Infections			
Genotype/Phenotype	No	Yes	Total	p-value[1]	No	Yes	Total	p-value[2]	No	Yes	Total	p-value[1]	No	Yes	Total	p-value[2]
PM	64 (58%)	47 (42%)	111 (100%)		102 (92%)	9 (8.1%)	111 (100%)		66 (59%)	45 (41%)	111 (100%)		104 (94%)	7 (6.3%)	111 (100%)	
Gain.1p				0.8				0.11				0.6				0.6
No	66 (59%)	45 (41%)	111 (100%)		104 (94%)	7 (6.3%)	111 (100%)		69 (62%)	42 (38%)	111 (100%)		104 (94%)	7 (6.3%)	111 (100%)	
Yes	9 (56%)	7 (44%)	16 (100%)		13 (81%)	3 (19%)	16 (100%)		9 (56%)	7 (44%)	16 (100%)		16 (100%)	0 (0%)	16 (100%)	
P53				0.2				0.15				0.3				>0.9
No	72 (61%)	46 (39%)	118 (100%)		110 (93%)	8 (6.8%)	118 (100%)		74 (63%)	44 (37%)	118 (100%)		111 (94%)	7 (5.9%)	118 (100%)	
Yes	3 (33%)	6 (67%)	9 (100%)		7 (78%)	2 (22%)	9 (100%)		4 (44%)	5 (56%)	9 (100%)		9 (100%)	0 (0%)	9 (100%)	
FGFR3				>0.9				0.4				>0.9				>0.9
No	71 (59%)	49 (41%)	120 (100%)		111 (93%)	9 (7.5%)	120 (100%)		74 (62%)	46 (38%)	120 (100%)		113 (94%)	7 (5.8%)	120 (100%)	
Yes	4 (57%)	3 (43%)	7 (100%)		6 (86%)	1 (14%)	7 (100%)		4 (57%)	3 (43%)	7 (100%)		7 (100%)	0 (0%)	7 (100%)	
MAF				>0.9				>0.9				>0.9				>0.9
No	74 (59%)	52 (41%)	126 (100%)		116 (92%)	10 (7.9%)	126 (100%)		77 (61%)	49 (39%)	126 (100%)		119 (94%)	7 (5.6%)	126 (100%)	
Yes	1 (100%)	0 (0%)	1 (100%)		1 (100%)	0 (0%)	1 (100%)		1 (100%)	0 (0%)	1 (100%)		1 (100%)	0 (0%)	1 (100%)	
Delection.1q				>0.9				>0.9				>0.9				>0.9
No	74 (59%)	51 (41%)	125 (100%)		115 (92%)	10 (8.0%)	125 (100%)		77 (62%)	48 (38%)	125 (100%)		118 (94%)	7 (5.6%)	125 (100%)	
Yes	1 (50%)	1 (50%)	2 (100%)		2 (100%)	0 (0%)	2 (100%)		1 (50%)	1 (50%)	2 (100%)		2 (100%)	0 (0%)	2 (100%)	

	Neurotoxicity				Neurotoxicity different from peripheral neuropathy				Peripheral neuropathy				Infections			
Genotype/Phenotype	No	Yes	Total	p-value[1]	No	Yes	Total	p-value[2]	No	Yes	Total	p-value[1]	No	Yes	Total	p-value[2]
CCND1				>0.9				0.2				0.5				>0.9
No	74 (59%)	51 (41%)	125 (100%)		116 (93%)	9 (7.2%)	125 (100%)		76 (61%)	49 (39%)	125 (100%)		118 (94%)	7 (5.6%)	125 (100%)	
Yes	1 (50%)	1 (50%)	2 (100%)		1 (50%)	1 (50%)	2 (100%)		2 (100%)	0 (0%)	2 (100%)		2 (100%)	0 (0%)	2 (100%)	
MM.double.hit				>0.9				0.12				0.5				>0.9
No	70 (59%)	49 (41%)	119 (100%)		111 (93%)	8 (6.7%)	119 (100%)		72 (61%)	47 (39%)	119 (100%)		112 (94%)	7 (5.9%)	119 (100%)	
Yes	5 (63%)	3 (38%)	8 (100%)		6 (75%)	2 (25%)	8 (100%)		6 (75%)	2 (25%)	8 (100%)		8 (100%)	0 (0%)	8 (100%)	

[1]Pearson's Chi-squared test

[2]Fisher's exact test

Incidence of ADRs according to genotype/phenotype

	Toxicity of metabolism and nutrition				Psychiatric				General				Ocular			
Genotype/Phenotype	No	Yes	Total	p-value[1]	No	Yes	Total	p-value[1]	No	Yes	Total	p-value[2]	No	Yes	Total	p-value[1]
*ABCB1*_C1236T (rs1128503)				0.5				0.5				0.3				0.7
A/A	22 (100%)	0 (0%)	22 (100%)		21 (95%)	1 (4.5%)	22 (100%)		15 (68%)	7 (32%)	22 (100%)		22 (100%)	0 (0%)	22 (100%)	
A/G	59 (100%)	0 (0%)	59 (100%)		56 (95%)	3 (5.1%)	59 (100%)		30 (51%)	29 (49%)	59 (100%)		56 (95%)	3 (5.1%)	59 (100%)	
G/G	44 (98%)	1 (2.2%)	45 (100%)		40 (89%)	5 (11%)	45 (100%)		23 (51%)	22 (49%)	45 (100%)		44 (98%)	1 (2.2%)	45 (100%)	
*ABCB1*_C3435T (rs1045642)				0.5				0.3				0.6				0.2
A/A	22 (100%)	0 (0%)	22 (100%)		22 (100%)	0 (0%)	22 (100%)		14 (64%)	8 (36%)	22 (100%)		20 (91%)	2 (9.1%)	22 (100%)	
A/G	68 (100%)	0 (0%)	68 (100%)		63 (93%)	5 (7.4%)	68 (100%)		35 (51%)	33 (49%)	68 (100%)		66 (97%)	2 (2.9%)	68 (100%)	
G/G	35 (97%)	1 (2.8%)	36 (100%)		32 (89%)	4 (11%)	36 (100%)		19 (53%)	17 (47%)	36 (100%)		36 (100%)	0 (0%)	36 (100%)	
*ABCB1*_G2677AT (rs2032582)				0.5				0.4				0.4				>0.9
A/A	16 (100%)	0 (0%)	16 (100%)		16 (100%)	0 (0%)	16 (100%)		11 (69%)	5 (31%)	16 (100%)		16 (100%)	0 (0%)	16 (100%)	
C/A_C/T	63 (100%)	0 (0%)	63 (100%)		59 (94%)	4 (6.3%)	63 (100%)		33 (52%)	30 (48%)	63 (100%)		61 (97%)	2 (3.2%)	63 (100%)	
C/C	46 (98%)	1 (2.1%)	47 (100%)		42 (89%)	5 (11%)	47 (100%)		24 (51%)	23 (49%)	47 (100%)		45 (96%)	2 (4.3%)	47 (100%)	

	Toxicity of metabolism and nutrition				Psychiatric				General				Ocular			
Genotype/Phenotype	No	Yes	Total	p-value[1]	No	Yes	Total	p-value[1]	No	Yes	Total	p-value[2]	No	Yes	Total	p-value[1]
CYP1A2				>0.9				0.5				0.5				>0.9
NM	58 (100%)	0 (0%)	58 (100%)		55 (95%)	3 (5.2%)	58 (100%)		30 (52%)	28 (48%)	58 (100%)		56 (97%)	2 (3.4%)	58 (100%)	
PM	1 (100%)	0 (0%)	1 (100%)		1 (100%)	0 (0%)	1 (100%)		0 (0%)	1 (100%)	1 (100%)		1 (100%)	0 (0%)	1 (100%)	
UM	66 (99%)	1 (1.5%)	67 (100%)		61 (91%)	6 (9.0%)	67 (100%)		38 (57%)	29 (43%)	67 (100%)		65 (97%)	2 (3.0%)	67 (100%)	
CYP2B6				>0.9				0.4				0.7				0.2
IM	47 (100%)	0 (0%)	47 (100%)		45 (96%)	2 (4.3%)	47 (100%)		26 (55%)	21 (45%)	47 (100%)		44 (94%)	3 (6.4%)	47 (100%)	
NM	58 (98%)	1 (1.7%)	59 (100%)		54 (92%)	5 (8.5%)	59 (100%)		30 (51%)	29 (49%)	59 (100%)		59 (100%)	0 (0%)	59 (100%)	
PM	15 (100%)	0 (0%)	15 (100%)		14 (93%)	1 (6.7%)	15 (100%)		8 (53%)	7 (47%)	15 (100%)		14 (93%)	1 (6.7%)	15 (100%)	
RM	5 (100%)	0 (0%)	5 (100%)		4 (80%)	1 (20%)	5 (100%)		4 (80%)	1 (20%)	5 (100%)		5 (100%)	0 (0%)	5 (100%)	
CYP2C19				0.3				0.2				0.5				0.5
IM	32 (100%)	0 (0%)	32 (100%)		29 (91%)	3 (9.4%)	32 (100%)		20 (63%)	12 (38%)	32 (100%)		31 (97%)	1 (3.1%)	32 (100%)	
NM	60 (100%)	0 (0%)	60 (100%)		58 (97%)	2 (3.3%)	60 (100%)		32 (53%)	28 (47%)	60 (100%)		59 (98%)	1 (1.7%)	60 (100%)	
PM	1 (100%)	0 (0%)	1 (100%)		1 (100%)	0 (0%)	1 (100%)		0 (0%)	1 (100%)	1 (100%)		1 (100%)	0 (0%)	1 (100%)	
RM	28 (97%)	1 (3.4%)	29 (100%)		26 (90%)	3 (10%)	29 (100%)		15 (52%)	14 (48%)	29 (100%)		27 (93%)	2 (6.9%)	29 (100%)	

	Toxicity of metabolism and nutrition				Psychiatric				General				Ocular			
Genotype/Phenotype	No	Yes	Total	p-value[1]	No	Yes	Total	p-value[1]	No	Yes	Total	p-value[2]	No	Yes	Total	p-value[1]
UM	4 (100%)	0 (0%)	4 (100%)		3 (75%)	1 (25%)	4 (100%)		1 (25%)	3 (75%)	4 (100%)		4 (100%)	0 (0%)	4 (100%)	
CYP2C9				0.5				0.6				0.2				0.7
IM	56 (98%)	1 (1.8%)	57 (100%)		54 (95%)	3 (5.3%)	57 (100%)		30 (53%)	27 (47%)	57 (100%)		56 (98%)	1 (1.8%)	57 (100%)	
NM	65 (100%)	0 (0%)	65 (100%)		59 (91%)	6 (9.2%)	65 (100%)		34 (52%)	31 (48%)	65 (100%)		62 (95%)	3 (4.6%)	65 (100%)	
PM	4 (100%)	0 (0%)	4 (100%)		4 (100%)	0 (0%)	4 (100%)		4 (100%)	0 (0%)	4 (100%)		4 (100%)	0 (0%)	4 (100%)	
CYP2D6				>0.9				>0.9				0.9				>0.9
IM	41 (100%)	0 (0%)	41 (100%)		37 (90%)	4 (9.8%)	41 (100%)		23 (56%)	18 (44%)	41 (100%)		40 (98%)	1 (2.4%)	41 (100%)	
NM	68 (99%)	1 (1.4%)	69 (100%)		64 (93%)	5 (7.2%)	69 (100%)		38 (55%)	31 (45%)	69 (100%)		66 (96%)	3 (4.3%)	69 (100%)	
PM	9 (100%)	0 (0%)	9 (100%)		9 (100%)	0 (0%)	9 (100%)		4 (44%)	5 (56%)	9 (100%)		9 (100%)	0 (0%)	9 (100%)	
UM	7 (100%)	0 (0%)	7 (100%)		7 (100%)	0 (0%)	7 (100%)		3 (43%)	4 (57%)	7 (100%)		7 (100%)	0 (0%)	7 (100%)	
CYP3A4				>0.9				**0.031**				0.6				0.3
IM	10 (100%)	0 (0%)	10 (100%)		7 (70%)	3 (30%)	10 (100%)		6 (60%)	4 (40%)	10 (100%)		9 (90%)	1 (10%)	10 (100%)	
NM	114 (99%)	1 (0.9%)	115 (100%)		109 (95%)	6 (5.2%)	115 (100%)		62 (54%)	53 (46%)	115 (100%)		112 (97%)	3 (2.6%)	115 (100%)	
PM	1 (100%)	0 (0%)	1 (100%)		1 (100%)	0 (0%)	1 (100%)		0 (0%)	1 (100%)	1 (100%)		1 (100%)	0 (0%)	1 (100%)	
CYP3A5				>0.9				0.6				0.6				>0.9

	Toxicity of metabolism and nutrition				Psychiatric				General				Ocular			
Genotype/Phenotype	No	Yes	Total	p-value[1]	No	Yes	Total	p-value[1]	No	Yes	Total	p-value[2]	No	Yes	Total	p-value[1]
IM	15 (100%)	0 (0%)	15 (100%)		15 (100%)	0 (0%)	15 (100%)		9 (60%)	6 (40%)	15 (100%)		15 (100%)	0 (0%)	15 (100%)	
PM	110 (99%)	1 (0.9%)	111 (100%)		102 (92%)	9 (8.1%)	111 (100%)		59 (53%)	52 (47%)	111 (100%)		107 (96%)	4 (3.6%)	111 (100%)	
Gain.1p				>0.9				0.3				0.7				>0.9
No	110 (99%)	1 (0.9%)	111 (100%)		104 (94%)	7 (6.3%)	111 (100%)		61 (55%)	50 (45%)	111 (100%)		107 (96%)	4 (3.6%)	111 (100%)	
Yes	16 (100%)	0 (0%)	16 (100%)		14 (88%)	2 (13%)	16 (100%)		8 (50%)	8 (50%)	16 (100%)		16 (100%)	0 (0%)	16 (100%)	
P53				>0.9				0.5				0.7				**0.025**
No	117 (99%)	1 (0.8%)	118 (100%)		110 (93%)	8 (6.8%)	118 (100%)		65 (55%)	53 (45%)	118 (100%)		116 (98%)	2 (1.7%)	118 (100%)	
Yes	9 (100%)	0 (0%)	9 (100%)		8 (89%)	1 (11%)	9 (100%)		4 (44%)	5 (56%)	9 (100%)		7 (78%)	2 (22%)	9 (100%)	
FGFR3				>0.9				>0.9				0.7				>0.9
No	119 (99%)	1 (0.8%)	120 (100%)		111 (93%)	9 (7.5%)	120 (100%)		66 (55%)	54 (45%)	120 (100%)		116 (97%)	4 (3.3%)	120 (100%)	
Yes	7 (100%)	0 (0%)	7 (100%)		7 (100%)	0 (0%)	7 (100%)		3 (43%)	4 (57%)	7 (100%)		7 (100%)	0 (0%)	7 (100%)	
MAF				>0.9				>0.9				>0.9				>0.9
No	125 (99%)	1 (0.8%)	126 (100%)		117 (93%)	9 (7.1%)	126 (100%)		68 (54%)	58 (46%)	126 (100%)		122 (97%)	4 (3.2%)	126 (100%)	
Yes	1 (100%)	0 (0%)	1 (100%)		1 (100%)	0 (0%)	1 (100%)		1 (100%)	0 (0%)	1 (100%)		1 (100%)	0 (0%)	1 (100%)	
Delection.1q				>0.9				>0.9				0.2				>0.9

	Toxicity of metabolism and nutrition				Psychiatric				General				Ocular			
Genotype/Phenotype	No	Yes	Total	p-value[1]	No	Yes	Total	p-value[1]	No	Yes	Total	p-value[2]	No	Yes	Total	p-value[1]
No	124 (99%)	1 (0.8%)	125 (100%)		116 (93%)	9 (7.2%)	125 (100%)		69 (55%)	56 (45%)	125 (100%)		121 (97%)	4 (3.2%)	125 (100%)	
Yes	2 (100%)	0 (0%)	2 (100%)		2 (100%)	0 (0%)	2 (100%)		0 (0%)	2 (100%)	2 (100%)		2 (100%)	0 (0%)	2 (100%)	
CCND1				>0.9				>0.9				>0.9				0.062
No	124 (99%)	1 (0.8%)	125 (100%)		116 (93%)	9 (7.2%)	125 (100%)		68 (54%)	57 (46%)	125 (100%)		122 (98%)	3 (2.4%)	125 (100%)	
Yes	2 (100%)	0 (0%)	2 (100%)		2 (100%)	0 (0%)	2 (100%)		1 (50%)	1 (50%)	2 (100%)		1 (50%)	1 (50%)	2 (100%)	
MM.double.hit				>0.9				>0.9				0.14				0.2
No	118 (99%)	1 (0.8%)	119 (100%)		110 (92%)	9 (7.6%)	119 (100%)		67 (56%)	52 (44%)	119 (100%)		116 (97%)	3 (2.5%)	119 (100%)	
Yes	8 (100%)	0 (0%)	8 (100%)		8 (100%)	0 (0%)	8 (100%)		2 (25%)	6 (75%)	8 (100%)		7 (88%)	1 (13%)	8 (100%)	

[1]Fisher's exact test

[2]Pearson's Chi-squared test

Incidence of ADRs according to genotype/phenotype

	Ototoxicity				Cardiotoxicity				Vascular				Hepatotoxicity			
Genotype/Phenotype	No	Yes	Total	p-value[1]	No	Yes	Total	p-value[1]	No	Yes	Total	p-value[1]	No	Yes	Total	p-value[1]
*ABCB1*_C1236T (rs1128503)				0.8				0.4				>0.9				0.7
A/A	21 (95%)	1 (4.5%)	22 (100%)		22 (100%)	0 (0%)	22 (100%)		21 (95%)	1 (4.5%)	22 (100%)		22 (100%)	0 (0%)	22 (100%)	
A/G	58 (98%)	1 (1.7%)	59 (100%)		58 (98%)	1 (1.7%)	59 (100%)		57 (97%)	2 (3.4%)	59 (100%)		56 (95%)	3 (5.1%)	59 (100%)	
G/G	44 (98%)	1 (2.2%)	45 (100%)		42 (93%)	3 (6.7%)	45 (100%)		44 (98%)	1 (2.2%)	45 (100%)		44 (98%)	1 (2.2%)	45 (100%)	
*ABCB1*_C3435T (rs1045642)				0.052				>0.9				0.5				>0.9
A/A	20 (91%)	2 (9.1%)	22 (100%)		21 (95%)	1 (4.5%)	22 (100%)		21 (95%)	1 (4.5%)	22 (100%)		21 (95%)	1 (4.5%)	22 (100%)	
A/G	68 (100%)	0 (0%)	68 (100%)		66 (97%)	2 (2.9%)	68 (100%)		65 (96%)	3 (4.4%)	68 (100%)		66 (97%)	2 (2.9%)	68 (100%)	
G/G	35 (97%)	1 (2.8%)	36 (100%)		35 (97%)	1 (2.8%)	36 (100%)		36 (100%)	0 (0%)	36 (100%)		35 (97%)	1 (2.8%)	36 (100%)	
*ABCB1*_G2677AT (rs2032582)				0.15				0.3				0.6				0.8
A/A	15 (94%)	1 (6.3%)	16 (100%)		16 (100%)	0 (0%)	16 (100%)		15 (94%)	1 (6.3%)	16 (100%)		16 (100%)	0 (0%)	16 (100%)	
C/A_C/T	63 (100%)	0 (0%)	63 (100%)		62 (98%)	1 (1.6%)	63 (100%)		61 (97%)	2 (3.2%)	63 (100%)		60 (95%)	3 (4.8%)	63 (100%)	
C/C	45 (96%)	2 (4.3%)	47 (100%)		44 (94%)	3 (6.4%)	47 (100%)		46 (98%)	1 (2.1%)	47 (100%)		46 (98%)	1 (2.1%)	47 (100%)	

	Ototoxicity				Cardiotoxicity				Vascular				Hepatotoxicity			
Genotype/Phenotype	No	Yes	Total	p-value[1]	No	Yes	Total	p-value[1]	No	Yes	Total	p-value[1]	No	Yes	Total	p-value[1]
CYP1A2				0.6				0.4				0.6				0.4
NM	56 (97%)	2 (3.4%)	58 (100%)		55 (95%)	3 (5.2%)	58 (100%)		57 (98%)	1 (1.7%)	58 (100%)		55 (95%)	3 (5.2%)	58 (100%)	
PM	1 (100%)	0 (0%)	1 (100%)		1 (100%)	0 (0%)	1 (100%)		1 (100%)	0 (0%)	1 (100%)		1 (100%)	0 (0%)	1 (100%)	
UM	66 (99%)	1 (1.5%)	67 (100%)		66 (99%)	1 (1.5%)	67 (100%)		64 (96%)	3 (4.5%)	67 (100%)		66 (99%)	1 (1.5%)	67 (100%)	
CYP2B6				0.8				0.13				0.2				0.7
IM	45 (96%)	2 (4.3%)	47 (100%)		46 (98%)	1 (2.1%)	47 (100%)		45 (96%)	2 (4.3%)	47 (100%)		46 (98%)	1 (2.1%)	47 (100%)	
NM	58 (98%)	1 (1.7%)	59 (100%)		58 (98%)	1 (1.7%)	59 (100%)		58 (98%)	1 (1.7%)	59 (100%)		57 (97%)	2 (3.4%)	59 (100%)	
PM	15 (100%)	0 (0%)	15 (100%)		14 (93%)	1 (6.7%)	15 (100%)		15 (100%)	0 (0%)	15 (100%)		14 (93%)	1 (6.7%)	15 (100%)	
RM	5 (100%)	0 (0%)	5 (100%)		4 (80%)	1 (20%)	5 (100%)		4 (80%)	1 (20%)	5 (100%)		5 (100%)	0 (0%)	5 (100%)	
CYP2C19				0.8				0.2				0.5				0.8
IM	31 (97%)	1 (3.1%)	32 (100%)		31 (97%)	1 (3.1%)	32 (100%)		31 (97%)	1 (3.1%)	32 (100%)		31 (97%)	1 (3.1%)	32 (100%)	
NM	59 (98%)	1 (1.7%)	60 (100%)		58 (97%)	2 (3.3%)	60 (100%)		59 (98%)	1 (1.7%)	60 (100%)		57 (95%)	3 (5.0%)	60 (100%)	
PM	1 (100%)	0 (0%)	1 (100%)		1 (100%)	0 (0%)	1 (100%)		1 (100%)	0 (0%)	1 (100%)		1 (100%)	0 (0%)	1 (100%)	
RM	28 (97%)	1 (3.4%)	29 (100%)		29 (100%)	0 (0%)	29 (100%)		27 (93%)	2 (6.9%)	29 (100%)		29 (100%)	0 (0%)	29 (100%)	
UM	4 (100%)	0 (0%)	4 (100%)		3 (75%)	1 (25%)	4 (100%)		4 (100%)	0 (0%)	4 (100%)		4 (100%)	0 (0%)	4 (100%)	

	Ototoxicity				Cardiotoxicity				Vascular				Hepatotoxicity			
Genotype/Phenotype	No	Yes	Total	p-value[1]	No	Yes	Total	p-value[1]	No	Yes	Total	p-value[1]	No	Yes	Total	p-value[1]
CYP2C9				>0.9				0.7				>0.9				**0.016**
IM	56 (98%)	1 (1.8%)	57 (100%)		56 (98%)	1 (1.8%)	57 (100%)		55 (96%)	2 (3.5%)	57 (100%)		54 (95%)	3 (5.3%)	57 (100%)	
NM	63 (97%)	2 (3.1%)	65 (100%)		62 (95%)	3 (4.6%)	65 (100%)		63 (97%)	2 (3.1%)	65 (100%)		65 (100%)	0 (0%)	65 (100%)	
PM	4 (100%)	0 (0%)	4 (100%)		4 (100%)	0 (0%)	4 (100%)		4 (100%)	0 (0%)	4 (100%)		3 (75%)	1 (25%)	4 (100%)	
CYP2D6				0.4				0.5				0.2				0.2
IM	40 (98%)	1 (2.4%)	41 (100%)		41 (100%)	0 (0%)	41 (100%)		39 (95%)	2 (4.9%)	41 (100%)		41 (100%)	0 (0%)	41 (100%)	
NM	68 (99%)	1 (1.4%)	69 (100%)		65 (94%)	4 (5.8%)	69 (100%)		68 (99%)	1 (1.4%)	69 (100%)		66 (96%)	3 (4.3%)	69 (100%)	
PM	8 (89%)	1 (11%)	9 (100%)		9 (100%)	0 (0%)	9 (100%)		9 (100%)	0 (0%)	9 (100%)		8 (89%)	1 (11%)	9 (100%)	
UM	7 (100%)	0 (0%)	7 (100%)		7 (100%)	0 (0%)	7 (100%)		6 (86%)	1 (14%)	7 (100%)		7 (100%)	0 (0%)	7 (100%)	
CYP3A4				>0.9				>0.9				>0.9				>0.9
IM	10 (100%)	0 (0%)	10 (100%)		10 (100%)	0 (0%)	10 (100%)		10 (100%)	0 (0%)	10 (100%)		10 (100%)	0 (0%)	10 (100%)	
NM	112 (97%)	3 (2.6%)	115 (100%)		111 (97%)	4 (3.5%)	115 (100%)		111 (97%)	4 (3.5%)	115 (100%)		111 (97%)	4 (3.5%)	115 (100%)	
PM	1 (100%)	0 (0%)	1 (100%)		1 (100%)	0 (0%)	1 (100%)		1 (100%)	0 (0%)	1 (100%)		1 (100%)	0 (0%)	1 (100%)	
CYP3A5				>0.9				>0.9				>0.9				0.4
IM	15 (100%)	0 (0%)	15 (100%)		15 (100%)	0 (0%)	15 (100%)		15 (100%)	0 (0%)	15 (100%)		14 (93%)	1 (6.7%)	15 (100%)	

	Ototoxicity				Cardiotoxicity				Vascular				Hepatotoxicity			
Genotype/Phenotype	No	Yes	Total	p-value[1]	No	Yes	Total	p-value[1]	No	Yes	Total	p-value[1]	No	Yes	Total	p-value[1]
PM	108 (97%)	3 (2.7%)	111 (100%)		107 (96%)	4 (3.6%)	111 (100%)		107 (96%)	4 (3.6%)	111 (100%)		108 (97%)	3 (2.7%)	111 (100%)	
Gain.1p				>0.9				>0.9				0.4				>0.9
No	108 (97%)	3 (2.7%)	111 (100%)		107 (96%)	4 (3.6%)	111 (100%)		108 (97%)	3 (2.7%)	111 (100%)		107 (96%)	4 (3.6%)	111 (100%)	
Yes	16 (100%)	0 (0%)	16 (100%)		16 (100%)	0 (0%)	16 (100%)		15 (94%)	1 (6.3%)	16 (100%)		16 (100%)	0 (0%)	16 (100%)	
P53				>0.9				>0.9				0.3				0.3
No	115 (97%)	3 (2.5%)	118 (100%)		114 (97%)	4 (3.4%)	118 (100%)		115 (97%)	3 (2.5%)	118 (100%)		115 (97%)	3 (2.5%)	118 (100%)	
Yes	9 (100%)	0 (0%)	9 (100%)		9 (100%)	0 (0%)	9 (100%)		8 (89%)	1 (11%)	9 (100%)		8 (89%)	1 (11%)	9 (100%)	
FGFR3				>0.9				>0.9				>0.9				>0.9
No	117 (98%)	3 (2.5%)	120 (100%)		116 (97%)	4 (3.3%)	120 (100%)		116 (97%)	4 (3.3%)	120 (100%)		116 (97%)	4 (3.3%)	120 (100%)	
Yes	7 (100%)	0 (0%)	7 (100%)		7 (100%)	0 (0%)	7 (100%)		7 (100%)	0 (0%)	7 (100%)		7 (100%)	0 (0%)	7 (100%)	
MAF				>0.9				>0.9				>0.9				>0.9
No	123 (98%)	3 (2.4%)	126 (100%)		122 (97%)	4 (3.2%)	126 (100%)		122 (97%)	4 (3.2%)	126 (100%)		122 (97%)	4 (3.2%)	126 (100%)	
Yes	1 (100%)	0 (0%)	1 (100%)		1 (100%)	0 (0%)	1 (100%)		1 (100%)	0 (0%)	1 (100%)		1 (100%)	0 (0%)	1 (100%)	
Delection.1q				>0.9				>0.9				>0.9				>0.9
No	122 (98%)	3 (2.4%)	125 (100%)		121 (97%)	4 (3.2%)	125 (100%)		121 (97%)	4 (3.2%)	125 (100%)		121 (97%)	4 (3.2%)	125 (100%)	
Yes	2 (100%)	0 (0%)	2 (100%)		2 (100%)	0 (0%)	2 (100%)		2 (100%)	0 (0%)	2 (100%)		2 (100%)	0 (0%)	2 (100%)	

	Ototoxicity				Cardiotoxicity				Vascular				Hepatotoxicity			
Genotype/Phenotype	No	Yes	Total	p-value[1]	No	Yes	Total	p-value[1]	No	Yes	Total	p-value[1]	No	Yes	Total	p-value[1]
CCND1				>0.9				>0.9				>0.9				0.062
No	122 (98%)	3 (2.4%)	125 (100%)		121 (97%)	4 (3.2%)	125 (100%)		121 (97%)	4 (3.2%)	125 (100%)		122 (98%)	3 (2.4%)	125 (100%)	
Yes	2 (100%)	0 (0%)	2 (100%)		2 (100%)	0 (0%)	2 (100%)		2 (100%)	0 (0%)	2 (100%)		1 (50%)	1 (50%)	2 (100%)	
MM.double.hit				>0.9				>0.9				>0.9				0.2
No	116 (97%)	3 (2.5%)	119 (100%)		115 (97%)	4 (3.4%)	119 (100%)		115 (97%)	4 (3.4%)	119 (100%)		116 (97%)	3 (2.5%)	119 (100%)	
Yes	8 (100%)	0 (0%)	8 (100%)		8 (100%)	0 (0%)	8 (100%)		8 (100%)	0 (0%)	8 (100%)		7 (88%)	1 (13%)	8 (100%)	

[1]Fisher's exact test

Incidence of ADRs according to genotype/phenotype

	Musculoskeletal				Cutaneous				Nephrotoxicity				Reproductive toxicity			
Genotype/Phenotype	No	Yes	Total	p-value[1]	No	Yes	Total	p-value[1]	No	Yes	Total	p-value[1]	No	Yes	Total	p-value[1]
*ABCB1*_C1236T (rs1128503)				0.2				0.4				0.8				>0.9
A/A	22 (100%)	0 (0%)	22 (100%)		20 (91%)	2 (9.1%)	22 (100%)		20 (91%)	2 (9.1%)	22 (100%)		22 (100%)	0 (0%)	22 (100%)	
A/G	51 (86%)	8 (14%)	59 (100%)		46 (78%)	13 (22%)	59 (100%)		55 (93%)	4 (6.8%)	59 (100%)		58 (98%)	1 (1.7%)	59 (100%)	
G/G	40 (89%)	5 (11%)	45 (100%)		37 (82%)	8 (18%)	45 (100%)		41 (91%)	4 (8.9%)	45 (100%)		45 (100%)	0 (0%)	45 (100%)	
*ABCB1*_C3435T (rs1045642)				0.11				0.7				>0.9				0.5
A/A	22 (100%)	0 (0%)	22 (100%)		18 (82%)	4 (18%)	22 (100%)		21 (95%)	1 (4.5%)	22 (100%)		22 (100%)	0 (0%)	22 (100%)	
A/G	61 (90%)	7 (10%)	68 (100%)		57 (84%)	11 (16%)	68 (100%)		62 (91%)	6 (8.8%)	68 (100%)		68 (100%)	0 (0%)	68 (100%)	
G/G	30 (83%)	6 (17%)	36 (100%)		28 (78%)	8 (22%)	36 (100%)		33 (92%)	3 (8.3%)	36 (100%)		35 (97%)	1 (2.8%)	36 (100%)	
*ABCB1*_G2677AT (rs2032582)				0.4				0.9				>0.9				0.5
A/A	16 (100%)	0 (0%)	16 (100%)		14 (88%)	2 (13%)	16 (100%)		15 (94%)	1 (6.3%)	16 (100%)		16 (100%)	0 (0%)	16 (100%)	
C/A_C/T	56 (89%)	7 (11%)	63 (100%)		51 (81%)	12 (19%)	63 (100%)		58 (92%)	5 (7.9%)	63 (100%)		63 (100%)	0 (0%)	63 (100%)	
C/C	41 (87%)	6 (13%)	47 (100%)		38 (81%)	9 (19%)	47 (100%)		43 (91%)	4 (8.5%)	47 (100%)		46 (98%)	1 (2.1%)	47 (100%)	

	Musculoskeletal				Cutaneous				Nephrotoxicity				Reproductive toxicity			
Genotype/Phenotype	No	Yes	Total	p-value[1]	No	Yes	Total	p-value[1]	No	Yes	Total	p-value[1]	No	Yes	Total	p-value[1]
CYP1A2				0.2				0.2				0.8				0.5
NM	55 (95%)	3 (5.2%)	58 (100%)		49 (84%)	9 (16%)	58 (100%)		54 (93%)	4 (6.9%)	58 (100%)		57 (98%)	1 (1.7%)	58 (100%)	
PM	1 (100%)	0 (0%)	1 (100%)		0 (0%)	1 (100%)	1 (100%)		1 (100%)	0 (0%)	1 (100%)		1 (100%)	0 (0%)	1 (100%)	
UM	57 (85%)	10 (15%)	67 (100%)		54 (81%)	13 (19%)	67 (100%)		61 (91%)	6 (9.0%)	67 (100%)		67 (100%)	0 (0%)	67 (100%)	
CYP2B6				0.5				0.2				0.4				0.5
IM	40 (85%)	7 (15%)	47 (100%)		37 (79%)	10 (21%)	47 (100%)		44 (94%)	3 (6.4%)	47 (100%)		46 (98%)	1 (2.1%)	47 (100%)	
NM	55 (93%)	4 (6.8%)	59 (100%)		52 (88%)	7 (12%)	59 (100%)		53 (90%)	6 (10%)	59 (100%)		59 (100%)	0 (0%)	59 (100%)	
PM	13 (87%)	2 (13%)	15 (100%)		10 (67%)	5 (33%)	15 (100%)		15 (100%)	0 (0%)	15 (100%)		15 (100%)	0 (0%)	15 (100%)	
RM	5 (100%)	0 (0%)	5 (100%)		4 (80%)	1 (20%)	5 (100%)		4 (80%)	1 (20%)	5 (100%)		5 (100%)	0 (0%)	5 (100%)	
CYP2C19				0.6				0.5				0.3				0.3
IM	28 (88%)	4 (13%)	32 (100%)		27 (84%)	5 (16%)	32 (100%)		31 (97%)	1 (3.1%)	32 (100%)		32 (100%)	0 (0%)	32 (100%)	
NM	52 (87%)	8 (13%)	60 (100%)		50 (83%)	10 (17%)	60 (100%)		56 (93%)	4 (6.7%)	60 (100%)		60 (100%)	0 (0%)	60 (100%)	
PM	1 (100%)	0 (0%)	1 (100%)		1 (100%)	0 (0%)	1 (100%)		1 (100%)	0 (0%)	1 (100%)		1 (100%)	0 (0%)	1 (100%)	
RM	28 (97%)	1 (3.4%)	29 (100%)		23 (79%)	6 (21%)	29 (100%)		25 (86%)	4 (14%)	29 (100%)		28 (97%)	1 (3.4%)	29 (100%)	

	Musculoskeletal				Cutaneous				Nephrotoxicity				Reproductive toxicity			
Genotype/Phenotype	No	Yes	Total	p-value[1]	No	Yes	Total	p-value[1]	No	Yes	Total	p-value[1]	No	Yes	Total	p-value[1]
UM	4 (100%)	0 (0%)	4 (100%)		2 (50%)	2 (50%)	4 (100%)		3 (75%)	1 (25%)	4 (100%)		4 (100%)	0 (0%)	4 (100%)	
CYP2C9				0.2				0.3				0.4				0.5
IM	48 (84%)	9 (16%)	57 (100%)		47 (82%)	10 (18%)	57 (100%)		50 (88%)	7 (12%)	57 (100%)		56 (98%)	1 (1.8%)	57 (100%)	
NM	61 (94%)	4 (6.2%)	65 (100%)		54 (83%)	11 (17%)	65 (100%)		62 (95%)	3 (4.6%)	65 (100%)		65 (100%)	0 (0%)	65 (100%)	
PM	4 (100%)	0 (0%)	4 (100%)		2 (50%)	2 (50%)	4 (100%)		4 (100%)	0 (0%)	4 (100%)		4 (100%)	0 (0%)	4 (100%)	
CYP2D6				0.5				0.3				>0.9				>0.9
IM	38 (93%)	3 (7.3%)	41 (100%)		37 (90%)	4 (9.8%)	41 (100%)		38 (93%)	3 (7.3%)	41 (100%)		41 (100%)	0 (0%)	41 (100%)	
NM	59 (86%)	10 (14%)	69 (100%)		53 (77%)	16 (23%)	69 (100%)		63 (91%)	6 (8.7%)	69 (100%)		68 (99%)	1 (1.4%)	69 (100%)	
PM	9 (100%)	0 (0%)	9 (100%)		7 (78%)	2 (22%)	9 (100%)		8 (89%)	1 (11%)	9 (100%)		9 (100%)	0 (0%)	9 (100%)	
UM	7 (100%)	0 (0%)	7 (100%)		6 (86%)	1 (14%)	7 (100%)		7 (100%)	0 (0%)	7 (100%)		7 (100%)	0 (0%)	7 (100%)	
CYP3A4				>0.9				0.5				0.6				>0.9
IM	9 (90%)	1 (10%)	10 (100%)		7 (70%)	3 (30%)	10 (100%)		9 (90%)	1 (10%)	10 (100%)		10 (100%)	0 (0%)	10 (100%)	
NM	103 (90%)	12 (10%)	115 (100%)		95 (83%)	20 (17%)	115 (100%)		106 (92%)	9 (7.8%)	115 (100%)		114 (99%)	1 (0.9%)	115 (100%)	
PM	1 (100%)	0 (0%)	1 (100%)		1 (100%)	0 (0%)	1 (100%)		1 (100%)	0 (0%)	1 (100%)		1 (100%)	0 (0%)	1 (100%)	
CYP3A5				>0.9				>0.9				0.10				0.12

	Musculoskeletal				Cutaneous				Nephrotoxicity				Reproductive toxicity			
Genotype/Phenotype	No	Yes	Total	p-value[1]	No	Yes	Total	p-value[1]	No	Yes	Total	p-value[1]	No	Yes	Total	p-value[1]
IM	14 (93%)	1 (6.7%)	15 (100%)		13 (87%)	2 (13%)	15 (100%)		12 (80%)	3 (20%)	15 (100%)		14 (93%)	1 (6.7%)	15 (100%)	
PM	99 (89%)	12 (11%)	111 (100%)		90 (81%)	21 (19%)	111 (100%)		104 (94%)	7 (6.3%)	111 (100%)		111 (100%)	0 (0%)	111 (100%)	
Gain.1p				>0.9				0.3				>0.9				>0.9
No	99 (89%)	12 (11%)	111 (100%)		89 (80%)	22 (20%)	111 (100%)		102 (92%)	9 (8.1%)	111 (100%)		110 (99%)	1 (0.9%)	111 (100%)	
Yes	15 (94%)	1 (6.3%)	16 (100%)		15 (94%)	1 (6.3%)	16 (100%)		15 (94%)	1 (6.3%)	16 (100%)		16 (100%)	0 (0%)	16 (100%)	
P53				0.2				0.7				**0.023**				>0.9
No	107 (91%)	11 (9.3%)	118 (100%)		97 (82%)	21 (18%)	118 (100%)		111 (94%)	7 (5.9%)	118 (100%)		117 (99%)	1 (0.8%)	118 (100%)	
Yes	7 (78%)	2 (22%)	9 (100%)		7 (78%)	2 (22%)	9 (100%)		6 (67%)	3 (33%)	9 (100%)		9 (100%)	0 (0%)	9 (100%)	
FGFR3				>0.9				>0.9				>0.9				>0.9
No	107 (89%)	13 (11%)	120 (100%)		98 (82%)	22 (18%)	120 (100%)		110 (92%)	10 (8.3%)	120 (100%)		119 (99%)	1 (0.8%)	120 (100%)	
Yes	7 (100%)	0 (0%)	7 (100%)		6 (86%)	1 (14%)	7 (100%)		7 (100%)	0 (0%)	7 (100%)		7 (100%)	0 (0%)	7 (100%)	
MAF				>0.9				>0.9				>0.9				>0.9
No	113 (90%)	13 (10%)	126 (100%)		103 (82%)	23 (18%)	126 (100%)		116 (92%)	10 (7.9%)	126 (100%)		125 (99%)	1 (0.8%)	126 (100%)	
Yes	1 (100%)	0 (0%)	1 (100%)		1 (100%)	0 (0%)	1 (100%)		1 (100%)	0 (0%)	1 (100%)		1 (100%)	0 (0%)	1 (100%)	
Delection.1q				>0.9				>0.9				>0.9				>0.9

	Musculoskeletal				Cutaneous				Nephrotoxicity				Reproductive toxicity			
Genotype/Phenotype	No	Yes	Total	p-value[1]	No	Yes	Total	p-value[1]	No	Yes	Total	p-value[1]	No	Yes	Total	p-value[1]
No	112 (90%)	13 (10%)	125 (100%)		102 (82%)	23 (18%)	125 (100%)		115 (92%)	10 (8.0%)	125 (100%)		124 (99%)	1 (0.8%)	125 (100%)	
Yes	2 (100%)	0 (0%)	2 (100%)		2 (100%)	0 (0%)	2 (100%)		2 (100%)	0 (0%)	2 (100%)		2 (100%)	0 (0%)	2 (100%)	
CCND1				0.2				0.3				>0.9				>0.9
No	113 (90%)	12 (9.6%)	125 (100%)		103 (82%)	22 (18%)	125 (100%)		115 (92%)	10 (8.0%)	125 (100%)		124 (99%)	1 (0.8%)	125 (100%)	
Yes	1 (50%)	1 (50%)	2 (100%)		1 (50%)	1 (50%)	2 (100%)		2 (100%)	0 (0%)	2 (100%)		2 (100%)	0 (0%)	2 (100%)	
MM.double.hit				0.6				>0.9				0.5				>0.9
No	107 (90%)	12 (10%)	119 (100%)		97 (82%)	22 (18%)	119 (100%)		110 (92%)	9 (7.6%)	119 (100%)		118 (99%)	1 (0.8%)	119 (100%)	
Yes	7 (88%)	1 (13%)	8 (100%)		7 (88%)	1 (13%)	8 (100%)		7 (88%)	1 (13%)	8 (100%)		8 (100%)	0 (0%)	8 (100%)	

[1]Fisher's exact test

Supplementary Table S3: Detailed descriptive statistics from the Kaplan–Meier analyses.

Peripheral neuropathy	N	Media	95% CI lower	95% CI upper
CYP3A4 NM	43	172.23	95.176867	249.28825
CYP3A4 IM	4	34.00	7.314112	60.68589
CYP3A4 PM	1	54.00	54.00	54.00000

Respiratory toxicity	N	Media	95% CI lower	95% CI upper
CYP3A4 NM	32	152.5	58.705709	246.29429
CYP3A4 IM	3	17.0	4.569486	29.43051
